# Novel expression system based on enhanced permeability of *Vibrio natriegens* cells induced by D,D- carboxypeptidase overexpression

**DOI:** 10.1007/s11274-023-03723-z

**Published:** 2023-08-12

**Authors:** Ľubica Kormanová, Zdenko Levarski, Andrej Minich, Viktor Varga, Lenka Levarská, Eva Struhárňanská, Ján Turňa, Stanislav Stuchlík

**Affiliations:** 1 Faculty of Natural Sciences, Comenius University in Bratislava, Ilkovičova 6, Bratislava, 811 04 Slovak Republic; 2 Science Park, Comenius University in Bratislava, Ilkovičova 8, Bratislava, 811 04 Slovak Republic

**Keywords:** Extracellular production, D,D-carboxypeptidases, Recombinant proteins, *Vibrio natriegens*

## Abstract

*Vibrio natriegens* is a fast-growing, non-pathogenic marine bacterium with promising features for biotechnological applications such as high-level recombinant protein production or fast DNA propagation. A remarkable short generation time (< 10 min), robust proteosynthetic activity and versatile metabolism with abilities to utilise wide range of substrates contribute to its establishment as a future industrial platform for fermentation processes operating with high productivity.

D,D-carboxypeptidases are membrane-associated enzymes involved in peptidoglycan biosynthesis and cell wall formation. This study investigates the impact of overexpressed D,D-carboxypeptidases on membrane integrity and the increased leakage of intracellular proteins into the growth medium in *V. natriegens*. Our findings confirm that co-expression of these enzymes can enhance membrane permeability, thereby facilitating the transport of target proteins into the extracellular environment, without the need for secretion signals, tags, or additional permeabilization methods. Using only a single step IMAC chromatography, we were able to purify AfKatG, MDBP or *Taq* polymerase in total yields of 117.9 ± 56.0 mg/L, 36.5 ± 12.9 mg/L and 26.5 ± 6.0 mg/L directly from growth medium, respectively. These results demonstrate the feasibility of our *V. natriegen*s based system as a broadly applicable extracellular tag-less recombinant protein producer.

**Supplementary Information:**

The online version contains supplementary material available at 10.1007/s11274-023-03723-z.

## Introduction

Prokaryotic host systems such as *Escherichia coli* or *Bacillus subtilis* are widely used for protein overproduction and allow to obtain large quantities of recombinant proteins in a short time. When pursuing industrial production, there is a preference for bacterial hosts that are fast-growing, well-established, and cost-effective. Novel prokaryotic platform based on *V. natriegens* offers many advantages over widely used host systems for protein production at laboratory and industrial scales. *V. natriegens* is gram-negative, non-pathogenic bacterium with shorter doubling time than *E. coli* (< 10 min) (Payne et al. 1961). It has a rapid proteosynthesis and exceptionally high biomass specific consumption rate (*qs*) under aerobic and anaerobic conditions (Hoffart et al. 2017). Moreover, *V. natriegens* possesses a robust metabolism, utilise various substrates and grows rapidly on low-cost media (Kormanová et al. 2020). Moreover, *V. natriegens* offers great metabolic capacity for intracellular and potentially extracellular protein production than *E. coli* (Schwarz et al. 2022).

Extracellular secretion offers several advantages over intracellular production, including simplifying downstream processes and improving the biological activity, solubility, and stability. Additionally, an extracellular production strategy becomes crucial when dealing with the expression of toxic proteins (Yoon et al. 2010). Similar to *E. coli*, *V. natriegens* has limited secretion capacity and typically cannot produce recombinant proteins in sufficient quantities directly into the growth medium (Cornelis 2000; Yoon et al. 2010). One of the extracellular production strategies includes a specific translocation across inner cytoplasmatic membrane using a secretion signal and translocation machinery. Especially TSS1 and TSS2 have been widely used for secretion of recombinant proteins in *E. coli* (Korotkov et al. 2012). However, one of the main drawbacks are a limited capacity of these machineries, weak secretion signal or size of secreted proteins. When the transport mechanism reaches its capacity, proteins have a tendency to form insoluble aggregates. Moreover, the secretion signal typically exhibits a specific secretion affinity when attached to a different recombinant protein, therefore the extensive optimization process is required. In general, large multidomain intracellular proteins may encounter difficulties in crossing the membrane. Therefore, additional approaches are required to enhance outer membrane permeabilization, such as the co-expression of lysis-promoting proteins, the addition of supplements like glycine (Yang et al. 1998), Triton X-100 (Fu 2010) or using leaky L-form mutant strains (Gumpert et al. 1998; Pang et al. 2022). However, these methods are often not suitable for industrial applications and high-density cell production of recombinant proteins.

A study by Yang et al. (2019) reported an alternative approach to enhance the permeability of both the inner and outer membrane in *E. coli*. This approach involves the overexpression of specific membrane-associated D-alanyl-D-alanine-carboxypeptidases (PBPs). Penicillin-binding proteins (PBPs) are proteins associated with the cell membrane that play a crucial role in the biosynthesis of peptidoglycan (PG). Their primary function is to catalyse the final steps in PG biosynthesis. The difference in the number and specificity of PBPs among different bacterial species is the results of specific environment conditions and different PG biosynthesis mechanisms. PBPs could be categorized into two main subclasses: High Molecular Weight (HMW) PBPs and Low Molecular Weight (LMW) PBPs. HMW PBPs consists of multi-domain proteins responsible for PG polymerization and biosynthesis. According to the activity and structure of N-terminal domain, Class A and Class B of HMW PBPs were characterized (Gittins et al. 1994). Class A PBPs are bifunctional proteins which have glycosyltransferase activity responsible for elongation of uncross-linked glycan units and transpeptidase activity of C-terminal domain providing peptide cross-linking between two glycan chains. Class B PBPs plays an important role in the cell morphogenesis and cooperation with other main proteins in the cell cycle. The LMW PBPs, with their D-alanyl-D-alanine carboxypeptidase activity, also play an important role in PG biogenesis and cell wall assembling by maintaining PG crosslinking and stabilization of cell wall structure. However, the LMW PBPs are often not essential for cell viability and growth. Therefore, they were selectively targeted for the generation of deletion mutants, aiming to induce membranes with a certain degree of permeability while minimizing the loss in viability (Denome at al. 1999; Ghosh et al. 2008). The overexpression of certain LMW PBPs could potentially also weaken PG structures of the cell wall and membrane integrity (Yang et al. 2019).

In general, the extracellular secretion approach can offer time and cost-saving benefits. We have designed a simple system for enhancing permeability in *V. natriegens* to provide alternative approach for extracellular targeting of recombinant proteins without the need for fusion-tags or secretion signals. This system is based solely on the strategy of overexpressing D,D-carboxypeptidases, which promotes the non-selective leakage of proteins into the growth media in *V. natriegens*.

## Materials and methods

### Construction of recombinant plasmids

The genes representing D,D-carboxypeptidases PBP4 and PBP5/6 respectively (Table 1) were amplified from *V. natriegens* ATCC 14,048 genome by PCR with specific primers and inserted into plasmid pRSFDuet (Table 2). Both genes were placed under T5 and T7 promoter resulting in recombinant constructs pRSFDuet-T7-PBP4, pRSFDuet-T7-PBP5/6, pRSFDuet-T5-PBP4 and pRSFDuet-T5-PBP5/6.

**Table 1:** Basic characteristics of LMW PBPs in *V. natriegens* used in this work. LWM – low molecular weight

LMW D,D-carboxypeptidases in *V. natriegens 14,048™*					
Name	Alternative name	Gene	Size (bp)	Basic characteristics	GenBank
D,D-carboxypeptidase PBP4	Penicillin binding protein 4	*dacB*	1422	PG biosynthesis and protein degradation	ALR14728.1
D,D-carboxypeptidase PBP5/6	Penicillin binding protein 5/6	*dacA*	1179	PG biosynthesis and protein degradation	ALR16268.1

**Table 2:** Sequence of primers used for the construction of recombinant plasmids

Used primers	Sequence
PBP4 ch1-F (*BglII*)	*GGCC* *AGATCT* *atgcgtttacgttggtcc*
PBP4 ch1-R (*PvuI*)	*GGCC* *GATCG* *ctacttcttaggcatcgcctg*
PBP 5/6 ch1-F (*BamHI*)	*GGCC* *GGATCC* *atgaataaaaataagtttgtgaaatctattctc*
PBP 5/6 ch1-R (*NotI*)	*GGCC* *GCGGCCGC* *ttagaagaagctcttaactaacaatac*
T5-F (*NotI*)	GGCCGCGGCCGCAGCTTCATGCACAGTGAAATCATG
T5-R (*BglII*)	GGCCAGATCTTTTTACCTCCTAAAAGTTAAACAAAATTATTTC
T5-PBP 5/6 (*BglII*)	GGCCAGATCTatgaataaaaataagtttgtgaaatctattctc
T5-PBP5/6 (*XhoI*)	*GGCC* *CTCGAG* *ttagaagaagctcttaactaacaatac*
AfkatG-F	ATGATGCGTCAGGGTGG
AfkatG-R	CTCGAGGCGCAGACCTGC
T5-GFP-F (*EcoRI*)	GGCCGAATTCGACCCCAAGGGCGACACC
T5-GFP-R	ATGTTTTACCTCCTAAAAGTTAAACAAAATTATTTCTAG
GFP-F	GATGAGTTGTTTAAATGCTAGCAAAGGAGAAGAAGAACTTTTCAC
GFP-R	GGGGGAATTCTTATTTGTAGAGCTCATCCATGCATGTG

### Media and culture conditions

LB3 medium (10 g/L peptone, 5 g/L yeast extract, 30 g/L NaCl) was used for cultivation of all *V. natriegens* strains (*V. natriegens* Vmax, Synthetic Genomics®, La Jolla, CA and *V. natriegens* PF, Pfeifer et al. 2019). The cultivations were carried on in the shake flasks at 37 °C on a rotary shaker (200 rpm). The expression of recombinant protein was induced by the addition of 1 mM IPTG. At the point of induction, bacterial cultures (AfKatG and MDBP expression) were also supplemented with hemin to a final concentration of 0.005%. BHI medium (37 g/L) (Sigma) supplemented with v2 salts (204 mM NaCl, 4.2 mM KCl, and 23.14 mM MgCl_2_) was used for preparation of electrocompetent cells.

### DCW determination

For the determination of dry cell weight (DCW), 1 ml sample of bacterial culture was harvested by centrifuging at 10,000 x g for 10 min at 4 °C. The resulting pellet was then dried at 95 °C until a constant weight was achieved.

### Bacterial transformation

*V. natriegens* strains were transformed using a modified electroporation protocol (Weinstock et al. 2016). *V. natriegens* cells were grown in BHIv2 medium to OD_600_ = 0.5. Bacterial culture was incubated on ice for 15 min. Cells were harvested at 4305 x g at 4 °C. Bacterial pellet was washed with electroporation buffer (680 mM sucrose, 7 mM K_2_HPO_4_, pH 7.2) and centrifuged at 4305 x g at 4 °C for total of three times. The pellet was resuspended in residual electroporation buffer and diluted with additional buffer to reach optical density value at 600 nm within 18–20. Plasmid DNA (50–200 ng) was added to cells, gently mixed, and transferred to a chilled electroporation cuvette (0.1 cm gap, Bio-Rad). Transformation was carried on with following parameters: 800 V, 25 25 μF, 200 Ω using Gene Pulser™ (Bio-Rad). Cells were immediately incubated in pre-heated recovery LB3 medium supplemented with 680 mM sucrose and shaken at 28 °C for at least 2 h.

### GFP assay

*V. natriegens* strains, containing prepared recombinant plasmids, were cultivated in LB3 medium at 37 °C overnight. Shake flasks (50 ml) with fresh LB3 medium were inoculated to final concentration of 1.0% (v/v) for expression of recombinant GFP. After reaching OD_600_ 0.5, GFP expression was induced by adding ITPG to a final concentration of 1 mM. Fluorescence intensity was measured in black 96-well plate using Varioskan Flash (Thermo Scientific). Excitation and emission wavelengths were 488 and 533 nm, respectively.

### Enzyme activity assay

Specific catalase activity was determined as decrease of an absorbance at 240 nm in phosphate buffer pH 6.0 with 10 mM H_2_O_2_ as substrate. The kinetics was measured by UV-Vis Spectrophotometer (UV-1800, SHIMADZU) for 2 min with 1 s interval for absorbance measurement. Results were recorded using UV Probe Ver. 2.42 software.

Specific peroxidase activity was determined as increase of absorbance at 405 nm in phosphate buffer pH 4.5 with 2 mM H_2_O_2_ and 0.9 mM ATBS as a substrate. The kinetics were measured in the same manner as in the case of catalase activity.

### Outer membrane assay

*N*-Phenyl-α-naphthylamine (NPN) was used for determination of outer membrane permeability (Loh et al. 1984). Cells were harvested by centrifugation at room temperature at 1000 x g for 10 min. Pellet was washed in 5 mM HEPES pH 7.2 and cells were diluted at final OD_600_ = 0.5. NPN solution was added to the cell suspension to the final concentration of 40 μM. Samples were immediately measured in a black 96-well plate using Varioskan Flash (Thermo Scientific). Measurements were performed before induction (0 min) and after induction (360 min). The fluorescence intensity was measured at excitation and emission wavelengths of 350 and 420 nm, respectively.

### Inner membrane assay

Propidium iodide (PI) was used for determination of inner membrane permeability (Ma et al. 2020). Cells were harvested by centrifugation at 8935 x g for 10 min after induction of D,D-carboxypeptidase overexpression and the supernatant was carefully removed. Cells were washed in the wash buffer (5 mM HEPES, 5 mM glucose pH 7.2). After the second round of centrifugation at 8935 x g cells were resuspended in the wash buffer. PI was added to the final concentration of 5 μM and incubated in the dark, at room temperature for 30 min. Measurements were performed before induction (0 min) and after induction (240 min). The fluorescence intensity was measured using Varioskan Flash (Thermo Scientific) at excitation wavelength 535 nm and emission wavelength 617 nm in the black 96-well plate.

### SDS-PAGE analysis

Samples for SDS-PAGE analysis of cells containing targeted proteins were harvested by centrifugation, resuspended in TE buffer, loading buffer, and incubated at 95 °C for 10 min. Prepared samples were analysed in 12% SDS-polyacrylamide gel (Laemmli, 1974).

An internal control sample for Western blot analysis was prepared using the same procedure as the other samples. The internal control consisted of a sample obtained from a 24-hour intracellular expression of *Taq* polymerase, following the exact conditions applied to the tested samples.

For protein sample preparation from growth medium, 1 ml of medium was deprived of bacterial cells by previous centrifugation and precipitated using trichloroacetic acid (TCA)/acetone. A volume of 250 μl TCA was added and incubated at 4 °C for 10 min. The sample was centrifuged at 21,952 x g for 5 min, pellet was washed with 200 μl acetone twice and dried at 95 °C for 5–10 min. Samples for SDS-PAGE were prepared as described for cell protein samples.

### Sample preparation for affinity chromatography

Bacterial cells were harvested at 7650 x g at 4 °C for 20 min and resuspended in equilibration buffer (TrisHCl pH 8.0, 0.5 M NaCl). Cells were disrupted by ultrasound using homogenizer Sonopuls HD3200 with KE 76 probe (Bandelin). The cell debris was removed by centrifugation at 7650 x g at 4 °C. Soluble fraction was stored on ice.

For protein purification from the growth media, bacterial cells were also harvested at 7650 x g at 4 °C for 20 min. Medium was carefully separated from cell pellet and diluted with equilibration buffer in ratio 1:1. Prepared sample was stored on ice.

### Affinity chromatography

Recombinant proteins (AfKatG, MDBP, *Taq*, Mut*Taq*) were expressed in fusion with His-tag sequence and afterwards purified using affinity chromatography on ÄKTA Avant 25 FPLC system (GE Healthcare) with 1 ml HisTrap HP (GE Healthcare). At the beginning of the purification run, the column was washed with 5 column volumes (5 CV) of equilibration buffer (50 mM TrisHCl, pH 8.0, 0.5 M NaCl). Sample was loaded onto column using the pump at speed of 0.1-1 CV/min. Afterwards, the column was washed with equilibration buffer (10 CV) with 5% addition of elution buffer (50 mM Tris-HCl pH 8.0, 0.5 M NaCl, 0.5 M imidazole) to remove non-bound and non-specifically bound proteins. Elution buffer with 0.5 M imidazole was used to release bound His-tagged proteins. Samples were taken automatically by the fraction collector based on predefined parameters.

### Western blot analyses

Protein samples separated on SDS-PAGE gels were transferred to the polyvinylidene fluoride (PVDF) membrane. The semi-dry transfer was performed in Tris-glycine buffer with SDS and isopropanol using Biometra Flashblot (Analytik Jena GmbH). Parameters of the analysis were set to 15 V, 250 mA for 45 min. Subsequently, the PVDF membrane was incubated in specific antibody solution (mouse anti-His) (Invitrogen) with dilution of 1:1000 in a 1% skim milk solution at 4 °C overnight. Further, the membrane was washed in goat anti-mouse secondary antibody (1:10 000) in 1% skim milk solution (Goat Anti-Mouse IgG, Sigma Aldrich) for 1 h at room temperature. For signal visualisation, the membrane was washed in chemiluminescence solution using Clarity Max Western ECL Substrate kit (Bio-Rad) according to the protocol and signal was detected using ImageQuant LAS 500 chemiluminescence CCD camera (GE Healthcare).

### Statistical analyses

All experiments were prepared in triplicates. Data are displayed as a mean with standard deviations (SD). For statistical analysis the student *t* test was used, and a *P* value of < 0.05 was considered as statistically significant.

## Results

### Effect of D,D-carboxypeptidases PBP4 or PBP5/6 overexpression on *V. natriegens* biomass formation

For purpose of this work, we have applied two *V. natriegens* strains for the determination of D,D-carboxypeptidase overexpression impact on culture growth and biomass formation. *V. natriegens* Vmax host strain (Synthetic Genomics®; La Jolla, California) possesses an inserted T7 polymerase cassette and is compatible with the T7-based series of expression vectors. *V. natriegens* PF (Prophage-free), prepared in the work Pfeifer et al. (2019), contains deletions of predicted prophage loci. These deletions have a significant effect on robustness and production stability of the strain (Pfeifer et al. 2019). However, *V. natriegens* PF is lacking T7-polymerase cassette, therefore we applied vectors with a hybrid T5-*lac* promoter for overexpression of PBPs and other target proteins. In case of *V. natriegens* Vmax, we have applied T7 promoter and T7 expression system as a useful alternative for production of recombinant proteins.

**Fig. 1:**
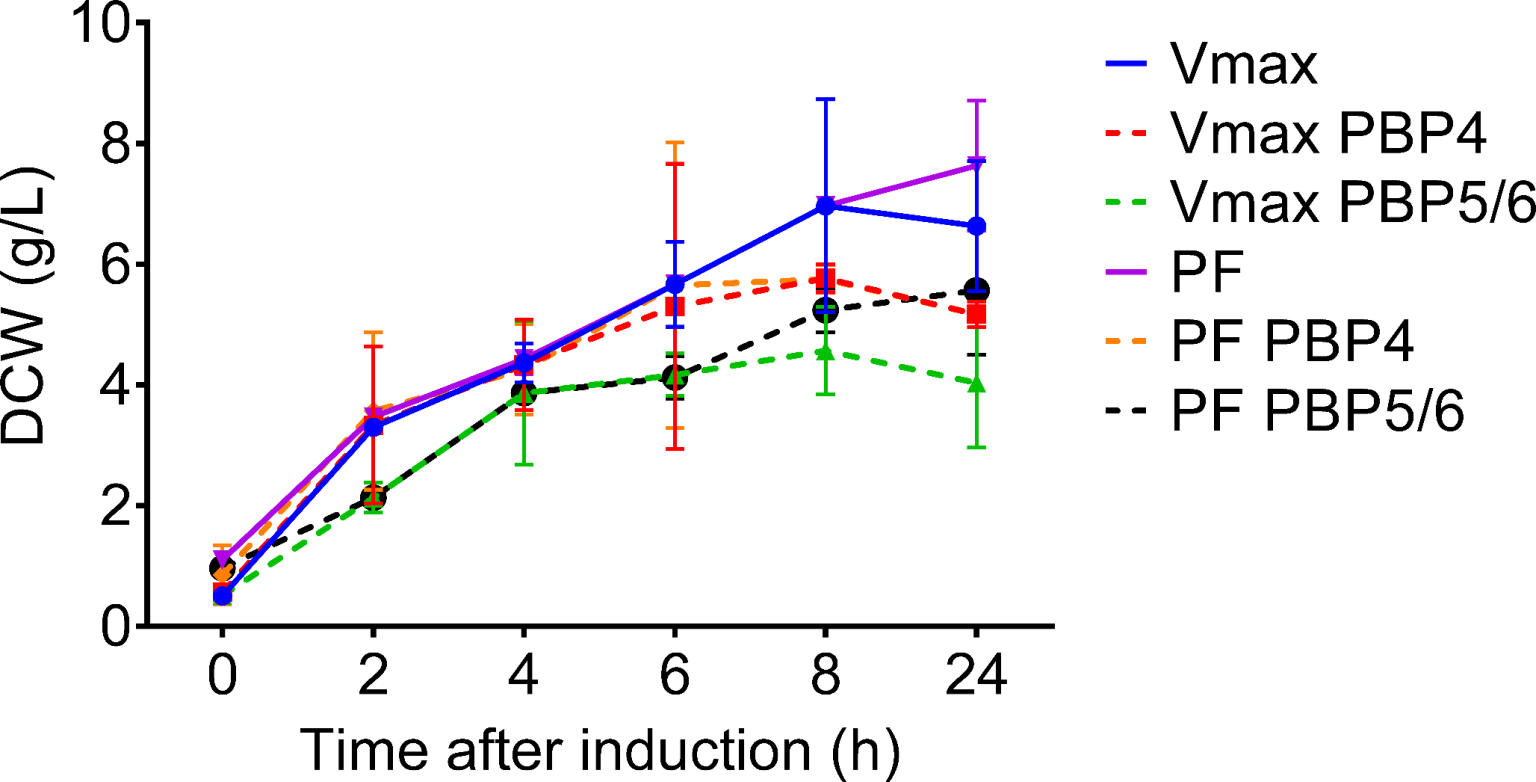
Determination of PBP4 or PBP5/6 D,D-carboxypeptidase overexpression on cell growth and biomass formation (dashed lines). Growth curves for *V. natriegens* Vmax and PF strains were determined according to DCW increase. D,D-carboxypeptidase overexpression was not present in control cultivations (Vmax and PF – solid lines). PF – *V. natriegens* Prophage-free; Vmax – *V. natriegens* Vmax; DCW - dry cell weight

First, we investigated the influence of D,D-carboxypeptidases PBP4 or PBP5/6 overexpression on *V. natriegens* growth. The decrease in a biomass formation was not statistically significant in all tested cultivations. However, the overexpression of PBP5/6 caused a stronger inhibition of biomass formation when overexpressed in both strains (Fig. 1). Moreover, we observed no significant impact on specific growth rate in *V. natriegens* Vmax or PF cultivations when PBP4 or PBP5/6 were overexpressed (See supplementary material). We also observed the increase of protein abundance in growth medium when PBP4 and PBP5/6 overexpressed in both *V. natriegens* strains. The strongest leak of intracellular protein was detected after 24 h of cultivation when compared to a control (See supplementary material).

### Determination of outer and inner membrane permeability in *V. natriegens* cells after D,D-carboxypeptidase PBP4 or PBP5/6 overexpression

**Fig. 2:**
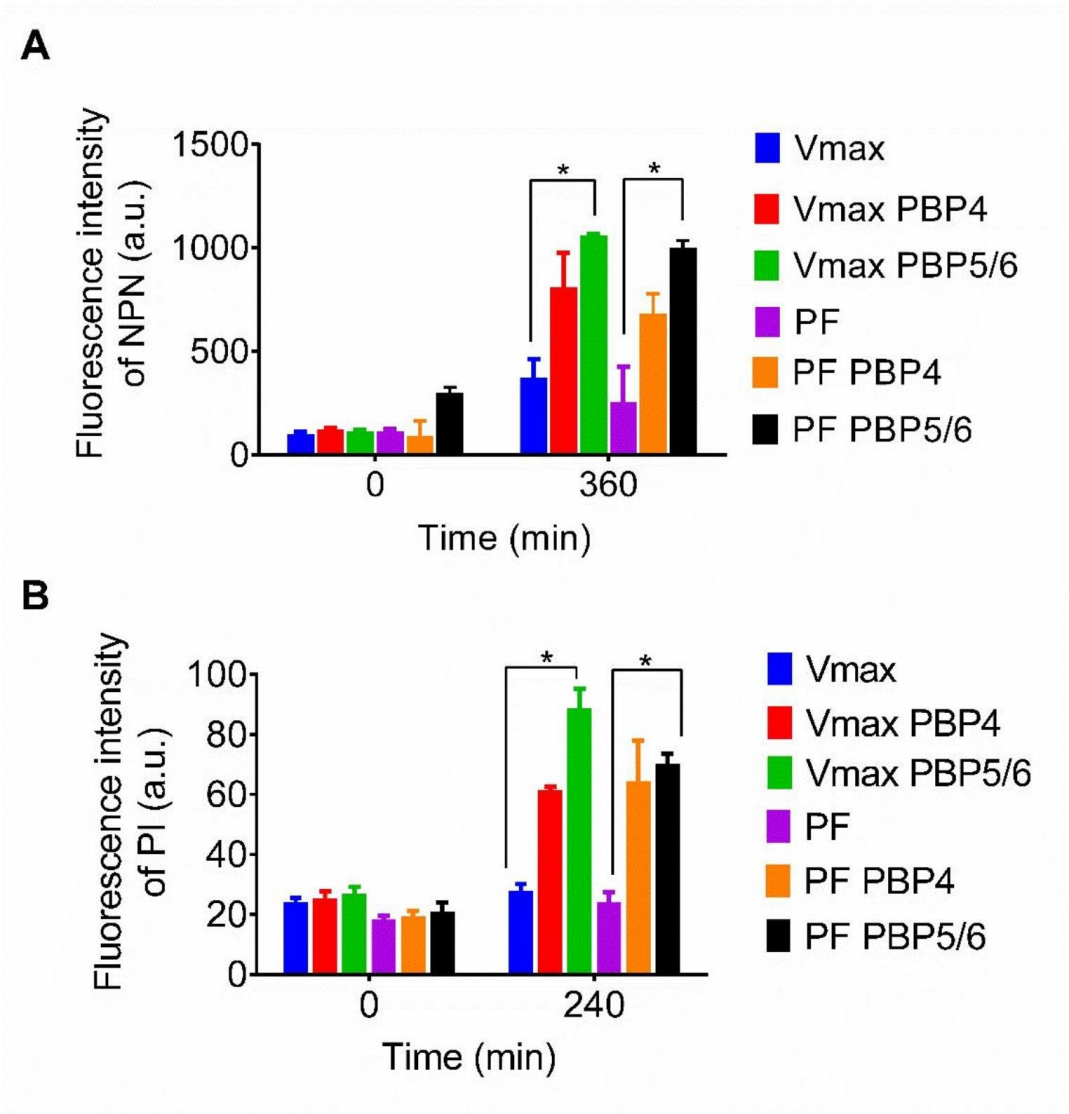
**A** - Outer membrane permeability of *V. natriegens* strains was determined by a fluorescence increase resulting from NPN uptake into the hydrophobic area of outer membrane. **B** - Inner membrane permeability of *V. natriegens* strains was determined by a fluorescence increase resulting from PI uptake into intracellular area of the cell. The cultivation without overexpression of PBP4 or PBP5/6 D,D-carboxypeptidases was used as a control. A *p*-value of less than 0.05 was considered statistically significant. PF – *V. natriegens* Prophage-free, Vmax – *V. natriegens* Vmax

NPN was used to detect outer membrane permeability. NPN fluorescence is in an aqueous environment weak, however it is highly increased in a nonpolar or hydrophobic environment. Overexpression of PBP5/6 caused an increase in fluorescent intensity in *V. natriegens* Vmax and *V. natriegens* PF in comparison with control (Fig. 2A). After PBP5/6 overexpression, we detected 2.8-fold increase of the fluorescence intensity in *V. natriegens* Vmax compared to control. Overexpression of PBP4 has no significant effect on outer cell membrane permeability in both *V. natriegens* strains (Fig. 2A).

PI is a red fluorescent nucleic acid dye and was used to detect inner membrane permeability. Overexpression of D,D-carboxypeptidase PBP5/6 caused the significant increase in PI fluorescent intensity in *V. natriegens* Vmax (by 3.2-fold) and in *V. natriegens* PF (by 2.5-fold) (Fig. 2B). Therefore, we assume that the overexpression of D,D-carboxypeptidase PBP5/6 might improve inner membrane permeability of *V. natriegens* strains.

### Overexpression of D,D-carboxypeptidase and its effect on extracellular GFP transport

**Fig. 3:**
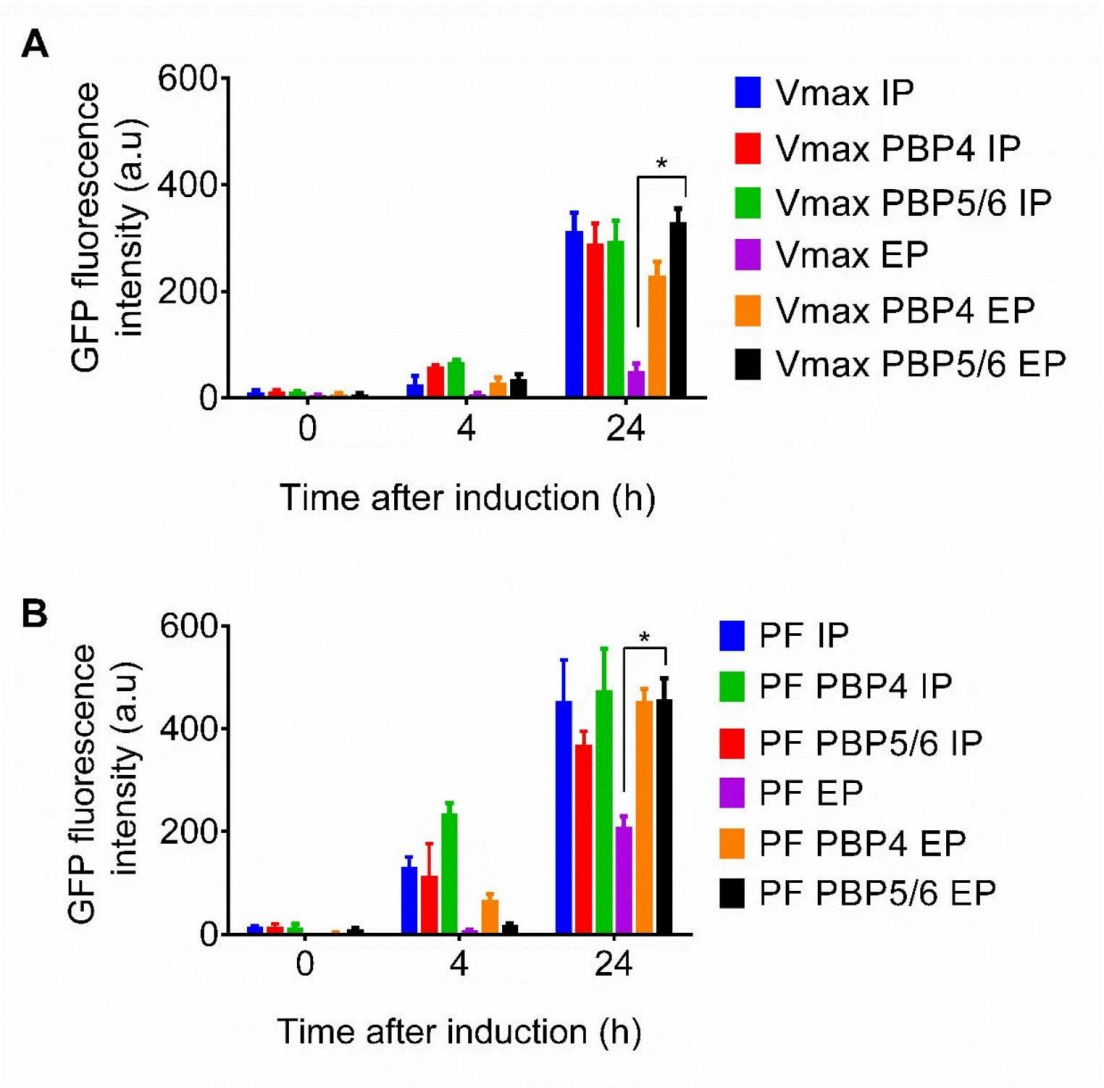
Effect of the D,D-carboxypeptidase PBP4 or PBP5/6 overexpression under T7-promoter on intracellular production of GFP as well as its extracellular transport into growth media in *V. natriegens* Vmax and PF **A** – Fluorescence intensity of intracellular and extracellular GFP with PBP4 or PBP5/6 co-expression after induction with 1 mM IPTG in *V. natriegens* Vmax; **B** – Fluorescence intensity of intracellular and extracellular GFP in medium after PBP4 or PBP5/6 co-expression after induction with 1 mM IPTG in *V. natriegens* PF; Control cultivations were lacking permeabilization plasmids for PBP4 and PBP5/6 overexpression. IP – Intracellular protein; EP – Extracellular protein; GFP - Green fluorescent protein; A *p*-value less than 0.05 was considered statistically significant. PF – *V. natriegens* Prophage-free; Vmax – *V. natriegens* Vmax

We also investigated an effect of co-expression of D,D-carboxypeptidases PBP4 or PBP5/6 with GFP and its influence on GFP leak into a growth media. We have not observed any significant differences between intracellular fluorescence intensity in *V. natriegens* Vmax (Fig. 3A) or *V. natriegens* PF (Fig. 3B). However, the fluorescence intensity in growth medium was increased in case of the overexpression both carboxypeptidases PBP4 and PBP5/6 in *V. natriegens* Vmax and *V. natriegens* PF. After PBP4 overexpression the fluorescence intensity increased by fold of 0.9 in *V. natriegens* Vmax and 4.5-fold in *V. natriegens* PF compared to control. In case of PBP5/6 overexpression the fluorescence intensity increased by fold of 6.4 in *V. natriegens* Vmax and 2.1-fold in *V. natriegens* PF compared to control.

### Overexpression of PBP5/6 enzyme and its effect on AfKatG extracellular transport

Thermostable catalase-peroxidase (AfKatG) is an enzyme originally isolated from hyperthermofillic archaeon *Archeoglobus fulgidus* (Beeder et al. 1994). It can be found in hydrothermal environment such as hot springs with the approximate temperature of 70–80 °C. Its catalase and peroxidase activity towards wide spectrum of substrates was in detail described. AfKatG is an interesting enzyme for a variety of industrial applications. From the structural point of view, it is a homodimer with subunit molecular mass of 85 kDa and contains binding site for a prosthetic group (ferric heme) in the stoichiometric ratio of 0.25 heme per subunit, crucial for its enzyme activities (Kengen et al. 2001).

**Fig. 4:**
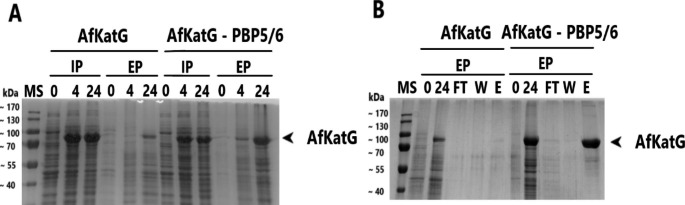
**A** - SDS-PAGE analysis of AfKatG production and its transport into growth medium in *V. natriegens* PF with co-expression of PBP5/6 under T5-*lac* promoter; **B** – SDS-PAGE analysis of AfKatG after IMAC purification from growth media. MS – protein molecular weight standard; IP – intracellular protein; EP – extracellular protein; FT – flow through; W- wash fraction; E – elution fraction; PF – *V. natriegens* Prophage-free; the numbering of lanes on SDS-PAGE or Western blot corresponds to the time in hours elapsed after induction

AfKatG was expressed under T5-*lac* promoter in *V. natriegens* PF to confirm its intracellular production and activity (Fig. 4A). Afterwards, AfKatG was used as a model protein for demonstration of intracellular protein leak into the growth medium using PBP5/6 D,D-carboxypeptidase co-expression in *V. natriegens* PF. When PBP5/6 co-expression was present, no significant decrease occurred in the intracellular production of AfKatG. However, a significant increase in extracellular AfKatG abundance occurred when the D,D-carboxypeptidase PBP5/6 was co-expressed (Fig. 4A). After purification from constant volume of growth medium using IMAC (at the same conditions) we were able to reach yields of 117.9 ± 56.0 mg/L when PBP5/6 co-expressed (Table 3). In control cultivation, the total gained yield of AfKatG purified from growth medium was 3.2 ± 1.3.

**Table 3:** Summary of purified AfKatG enzyme from different fractions of bacterial culture using affinity chromatography. IP – intracellular protein (without PBP5/6 overexpression); IP PBP5/6 – intracellular protein (with PBP5/6 overexpression); EP – extracellular protein (without PBP5/6 overexpression); EP PBP5/6 – extracellular protein (with PBP5/6 overexpression); the symbol “±” signifies the standard deviation associated with a measurement of three independent biological replicates

AfKatG				
	IP	IP PBP5/6	EP	EP PBP5/6
Protein yield (mg/L)	342.8 ± 34.3	293.5 ± 3.9	3.2 ± 1.3	117.9 ± 56.0
Enzyme-specific peroxidase activity (U/mg)	5.2 ± 1.8	4.5 ± 0.8	-	3.9 ± 1.1
Enzyme-specific catalase activity (U/mg)	1865 ± 203	1766.2 ± 155.7	-	1358.3 ± 247.2

Catalase and peroxidase activity was measured to confirm catalytic activity of AfKatG. No significant difference in catalase or peroxidase activity was detected when isolated from medium, cells or permeabilized cells (Table 3).

### Overexpression of PBP5/6 enzyme and its effect on MDBP peroxidase extracellular transport

MDPB (manganese-dependent bacterial peroxidase) enzyme belongs to the peroxidase family which was previously identified through bioinformatics analysis. The primary structure of the protein involves GXXDG motive which is specific for peroxidases and is also binding site for a prosthetic group (ferric heme). This enzyme is characterized by its ability to degrade lignin and cellulose, making it highly promising for various biotechnological applications.

**Fig. 5:**
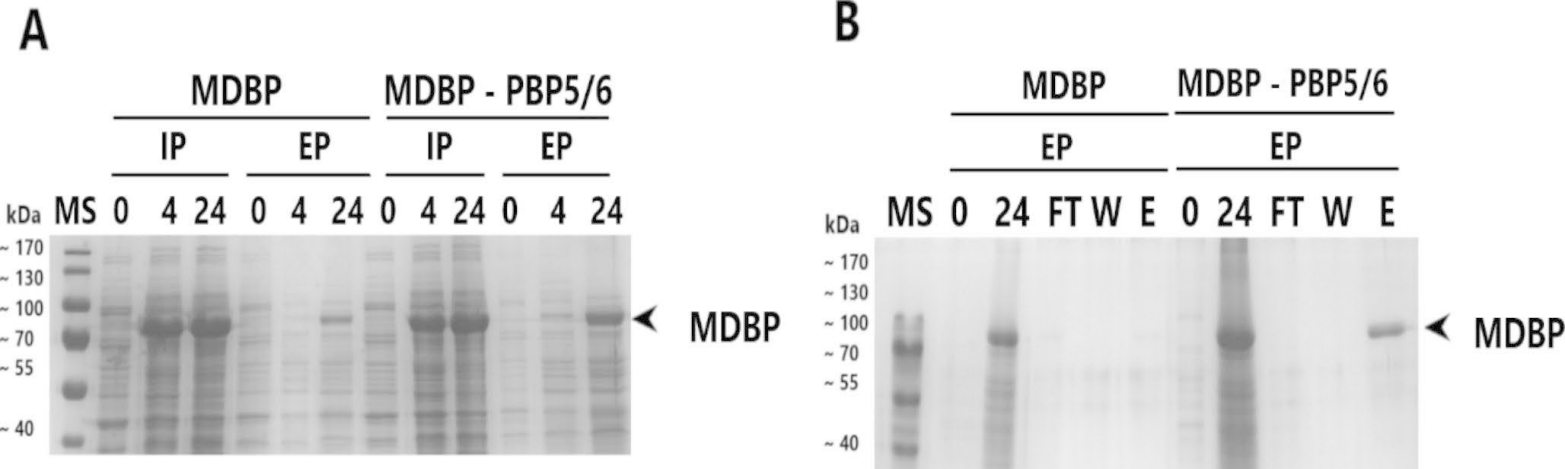
**A** - SDS-PAGE analysis of MDBP production and its extracellular transport into growth medium in *V. natriegens* PF with co-expression of PBP5/6 under T5-*lac* promoter; **B** – SDS-PAGE analysis of MDBP production after IMAC purification from growth medium. Control cultivation was lacking plasmid for D,D-carboxypeptidase overexpression. MS – protein molecular weight standard; IP – intracellular protein; EP – extracellular protein; the numbering of lanes on SDS-PAGE or Western blot corresponds to the time in hours elapsed after induction

MDBP was produced under T5-*lac* promoter in *V. natriegens* PF to investigate a co-expression effect of PBP5/6 and its transfer into growth medium (Fig. 5A). The level of intracellular production based on densitometric analysis was after 24 h ± 21% of TCP in control and ± 19% of TCP when PBP5/6 co-expressed. The soluble fraction of MDBP was calculated at the level of ± 31% and ± 33%, with and without co-expression of PBP5/6, respectively. Further, we also purified MDBP from cell fraction and medium using IMAC chromatography (Fig. 5B). We detected a minimal effect on MDBP intracellular production when PBP5/6 co-expressed. However, after purification of MDBP directly from growth medium, we detected a significant increase of MDBP in elution sample compared to control cultivations (Table 4). Total purified yield of MDBP from medium using one-step IMAC was 36.5 ± 12.9 mg/L resulting in increase compared to the control.

**Table 4:** Summary of purified *MDBP* enzyme from different fractions of bacterial culture using affinity chromatography. IP – intracellular protein (without PBP5/6 overexpression); IP PBP5/6 – intracellular protein (with PBP5/6 overexpression); EP – extracellular protein (without PBP5/6 overexpression); EP PBP5/6 – extracellular protein (with PBP5/6 overexpression); the symbol “±” signifies the standard deviation associated with a measurement of three independent biological replicates

MDBP				
	IP	IP PBP5/6	EP	EP PBP5/6
Protein yield (mg/L)	139.6 ± 34.0	120.0 ± 35.4	5.0 ± 0.02	36.5 ± 12.9
Enzyme-specific peroxidase activity (U/mg)	3.0 ± 0.3	2.48 ± 0.4	-	1.8 ± 0.3

Peroxidase activity was measured to confirm its catalytic activity (Table 4). No significant difference in peroxidase activity of MDBP isolated from medium, cells or permeabilized cells was observed.

### Overexpression of PBP5/6 enzyme and its effect on *Taq* and Mut*Taq* polymerase extracellular transport

DNA polymerases are often used for various genetic engineering techniques, including DNA cloning, mutagenesis, sequencing, and DNA labelling. One of the most widely used polymerases is a thermostable *Taq* polymerase from *Thermus aquaticus.* Protein engineering techniques were used to generate mutant enzymes, such as a cold-sensitive mutant of *Taq* polymerase or mutant *Taq* polymerase with a resistance to various PCR inhibitors including plasma, blood, hemoglobin or lactoferrin (Kermekchiev et al. 2003, 2009). The design of novel mutant enzymes could provide a promising alternative to the currently available commercial polymerases.

**Fig. 6:**
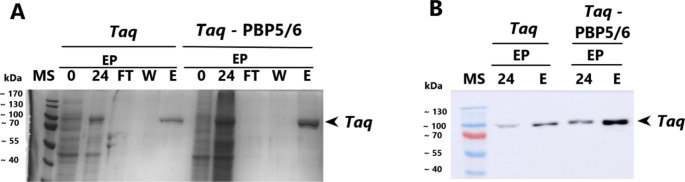
**A** - SDS-PAGE analysis of *Taq* polymerase production and its extracellular transport into growth medium in *V. natriegens* Vmax with co-expression of PBP5/6 under T7 promoter; **B** – Western blot analysis of *Taq* polymerase production after IMAC purification from growth medium. Control cultivations were lacking plasmid for PBP5/6 overexpression. MS – protein molecular weight standard; FT – flow through; W- wash fraction; E – elution fraction, IP – intracellular protein; EP – extracellular protein; Vmax – *V. natriegens* Vmax; the numbering of lanes on SDS-PAGE or Western blot corresponds to the time in hours elapsed after induction

**Fig. 7:**
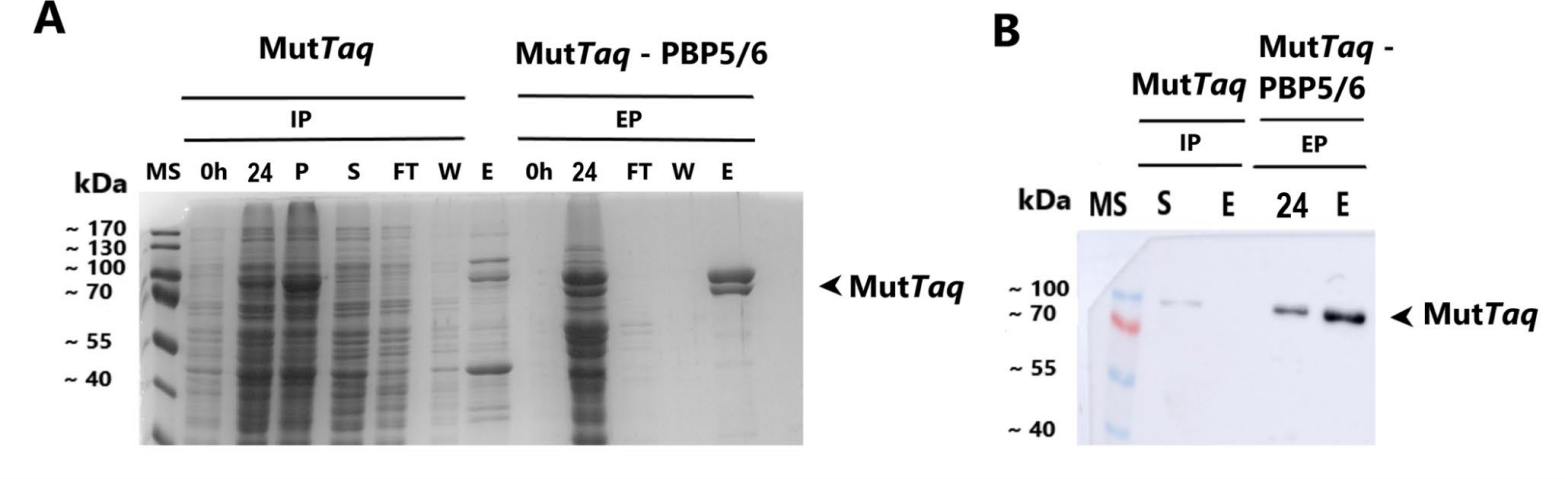
**A** - SDS-PAGE analysis of Mut*Taq* polymerase production and its extracellular transport into growth media in *V. natriegens* Vmax with co-expression of PBP5/6 under T7 promoter; **B** – Western blot analysis of Mut*Taq* polymerase production after IMAC purification from growth medium. Control cultivations were lacking plasmid for D,D-carboxypeptidase PBP5/6 overexpression. MS – protein molecular weight standard; P – insoluble fraction; S – soluble fraction of Mut*Taq* polymerase after 24 h intracellular production; FT – flow through; W- wash fraction; E – elution fraction, IP – intracellular protein; EP – extracellular protein; Vmax – *V. natriegens* Vmax; the numbering of lanes on SDS-PAGE or Western blot corresponds to the time in hours elapsed after induction

In this study, we expressed the native *Taq* polymerase (Fig. 6A) and a mutant *Taq* (Mut*Taq*) polymerase (Fig. 7A) with size of 94 kDa. The expression was carried out under the T7 promoter in *V. natriegens* Vmax. We compared the extracellular production of these enzymes using PBP5/6 co-expression approach to a control intracellular production (without PBP5/6 overexpression).

The co-expression of PBP5/6 enhanced the leak of intracellular proteins including already overexpressed wild type *Taq* polymerase into growth medium. Following affinity chromatography, the total yield of purified *Taq* polymerase directly obtained from the growth medium was approximately 26.5 ± 6.0 mg/L (Fig. 6B).

To enhance the beneficial properties of *Taq* polymerase for future PCR applications (ongoing research), specific mutations were introduced into its DNA sequence. However, these introduced mutations had a negative effect on its solubility (Fig. 7A). Mutated *Taq* polymerase (Mut*Taq*) was used as a model protein with a low solubility for demonstration of PBP5/6 co-expression strategy (Fig. 7A and B). The intracellular production of Mut*Taq* polymerase after 24 h was at level of 11% TCP and 8.7% TCP when PBP5/6 co-expressed. The soluble intracellular fraction was not observed after Western blot analysis (Fig. 7B). The significant higher amount of Mut*Taq* polymerase was observed only when purified from growth medium with co-expression of PBP5/6. We verified this also by Western blot analysis (Fig. 7B).

Both purified *Taq* and Mut*Taq* polymerases were lacking their enzymatic activity (See supplementary material). We incorporated an ammonium sulfate precipitation step in the downstream processing of the polymerases to eliminate any residual nucleic acids that might potentially affect the activity of these enzymes (Pluthero 1993). However, our attempts to obtain an active sample of *Taq* or Mut*Taq* polymerase were unsuccessful, indicating the need for further optimization (Table 5).

**Table 5:** Summary of purified *Taq* and Mut*Taq* enzymes from different fractions of bacterial culture using affinity chromatography. IP – intracellular protein (without PBP5/6 overexpression); IP PBP5/6 – intracellular protein (with PBP5/6 overexpression); EP – extracellular protein (without PBP5/6 overexpression); EP PBP5/6 – extracellular protein (with PBP5/6 overexpression); the symbol “±” signifies the standard deviation associated with a measurement of three independent biological replicates

***Taq***				
Protein yield (mg/L)	**IP**	**IP PBP5/6**	**EP**	**EP PBP5/6**
	17.6 ± 3.5	3.2 ± 3.0	1.0 ± 0.3	26.5 ± 6.0
**Mut** ***Taq***				
Protein yield (mg/L)	**IP**	**IP PBP5/6**	**EP**	**EP PBP5/6**
	-	-	-	10.4 ± 5.6

## Discussion

We investigated the impact of overexpressing D,D-carboxypeptidases PBP4 and PBP5/6 on the transport of model proteins into the growth media, as well as their influence on both outer and inner membrane permeability, biomass formation, and the potential for recombinant protein production. As previously mentioned, PBPs play an important role in PG biosynthesis, maturation, and maintaining the robustness of surface structures of the bacterial cell. We hypothesized that the homologues of D,D-carboxypeptidases within the LMW PBPs group, specific for *V. natriegens* strains, could affect the robustness and stability of surface structures without causing significant negative effects on viability or overall recombinant protein production. We focused especially on LMW PBPs because HMW PBPs are often crucial for cell viability and their deletion could result in the range of negative effects. For instance, the deficiency of either PBP1 or PBP2 of HMW PBPs in *E. coli* slows the growth, disrupts surface structures, changes the morphology of bacterial cell, and eventually results in complete cell lysis (Yousif et al. 1985; Suzuki 1978). However, the LMW PBP are often not essential for cell viability and survival. Extensive morphological aberrations and crucial dysfunctions were observed just in *E. coli* mutants lacking multiple LMW PBPs (Nelson et al. 2002). The deletion of the LMW PBP5 homologue, possessing D,D-carboxypeptidase activity, primarily affects cell shape and membrane stability in *E. coli*, as well as in other bacterial species such as *Bacillus subtilis*, *Listeria monocytogenes* or *Vibrio parahaemolyticus* (Korsak et al. 2005; Atrih et al. 1999; Hung et al. 2013). LMW PBPs with D,D-carboxypeptidase activity are crucial for removing terminal D-alanine from the pentapeptide chain, which facilitates and enhances PG crosslinking. The deletion or overexpression of these enzymes has a potential to cause an imbalance in PG biosynthesis, crosslinking, or bacterial cell stability. This can lead to greater leakage of intracellular proteins into the growth medium in *E. coli* (Hu et al. 2019; Yang et al. 2019).

We used the *V. natriegens* PF strain as a host for the overexpression of D,D-carboxypeptidases PBP4 and PBP5/6 and compared its growth pattern with the commercial host strain *V. natriegens* Vmax. The overexpression of D,D-carboxypeptidases PBP4 and PBP5/6 in both *V. natriegens* strains had no significant impact on biomass formation during cultivation, as we assumed based on previous research (Korsak et al. 2005; Atrih et al. 1999; Hung et al. 2013).

*V. natriegens* PF strain lacks two prophage loci in its genome and has potential advantages in terms of robustness against DNA damage and hypo-osmotic stress. Furthermore, this strain has demonstrated the capability to outcompete wild-type strains in growth and biomass formation (Pfeifer, 2019). These characteristics could be particularly useful in the context of D,D-carboxypeptidase overexpression, as they help minimize any negative effects on biomass production, culture viability, or growth inhibition. After 24 h of cultivation, we observed only a slight decrease in biomass when PBP5/6 was overexpressed in both *V. natriegens* strains. The growth curves were similar when D,D-carboxypeptidases were co-expressed, either in *V. natriegens* Vmax under the T7 promoter or in *V. natriegens* PF under the T5-*lac* promoter. No significant effect on biomass formation was observed when using *V. natriegens* PF strain. Therefore, we decided to use both strains equally as alternative host platforms (Fig. 1).

Next, we have analysed the outer and inner membrane permeability in both *V. natriegens* strains. After overexpression of LMW PBP5/6, a noticeable increase in fluorescence intensity of NPN was observed after 360 min of incubation, both in *V. natriegens* PF and *V. natriegens* Vmax (Fig. 2). This suggests the potential disruption of the outer membrane, facilitating the entry of NPN molecules into the hydrophobic periplasm. We also investigated the complexity of the inner membrane using PI dye, since it can only label bacteria with highly disrupted inner membrane. A significant PI fluorescence was observed after PBP5/6 overexpression in both host strains after 280 min of cultivation, and after 360 min of cultivation only in *V. natriegens* PF (Fig. 2). The observed increase in inner and outer membrane permeability might be the result of PG formation issues or disruption of the cell membrane.

The analysis of SDS-PAGE revealed the increase in the overall abundance of total proteins in the growth medium after induction of D,D-carboxypeptidase overexpression. The overall abundance of proteins in the medium was highest after 24 h of cultivation in both *V. natriegens* Vmax and *V. natriegens* PF strains (See supplementary material). To validate this observation, we performed co-expression experiments involving a model GFP protein with each D,D-carboxypeptidase in *V. natriegens* PF and *V. natriegens* Vmax. No significant change in fluorescence intensity was observed for intracellular production (Fig. 3). However, a significant increase in fluorescence intensity was observed in the growth medium compared to the control without co-expression of D,D-carboxypeptidases, particularly after 24 h of cultivation (Fig. 3). Therefore, we performed all subsequent cultivations within a 24-hour timeframe to achieve the highest possible abundance of intracellular model proteins in growth medium.

Subsequently, we performed a co-expression of PBP5/6 D,D-carboxypeptidase in *V. natriegens* PF to produce the heme protein AfKatG directly into the growth medium. We were able to achieve significantly higher yields of AfKatG in medium compared to the extracellular control of AfKatG without PBP5/6 co-expression. The overall production of AfKatG from medium reached a level of 117.9 ± 56.0 mg/L, which was significantly higher than the control without co-expression of PBP5/6. This alternative approach offers additional advantages by simplifying downstream processes and eliminating the need for homogenization steps. We also observed a small amount of AfKatG in the extracellular fraction of control culture after 24 h. In studies involving Gram-negative bacteria, centrifugation forces exceeding 5000 x g can result in cell disruption or the presence of the protein in medium can be simply the result of natural cell death and disruption during the cultivation cycle. For all downstream processes, we used a protocol using a higher centrifugation force (21,952 x g), which could potentially cause an additional spontaneous lysis and release of AfKatG from cells. However, the same protocol was consistently applied for preparation of all samples including the control (Pembrey et al. 1999).

After 24 h of cultivation, the intracellular abundance of AfKatG decreased by 16% when D,D-carboxypeptidase PBP5/6 was co-expressed, potentially due to the transport of AfKatG into the growth medium. However, no significant change in overall AfKatG concentration from soluble intracellular fraction was detected. This could be due to the shift of the solubility ratio because of lower protein concentration in the cell (due to intracellular protein leakage). Thus, the originally insoluble fraction could in this case replace the AfKatG already transported into the growth medium. The reduction of cellular protein concentration could enhance folding and solubility of protein. Previous studies have reported methods for reducing cellular protein abundance such as using low induction levels of inductor or weak promoter for solubility increase (Sørensen et al. 2005). This explanation was also further supported by densitometric analysis of the soluble fraction, indicating a change in the solubility ratio when comparing AfKatG production with and without D,D-carboxypeptidase PBP5/6 co-expression.

We have further evaluated the system by expressing MDBP heme peroxidase. We observed an increase in overall MDBP extracellular yields by fold of 6.8 when PBP5/6 co-expressed in *V. natriegens* PF compared to the control (Fig. 5). No significant changes in intracellular production were detected due to solubility shift, similar to AfKatG production.

At the time of induction, all AfKatG and MDBP cultures were supplemented with hemin as the source of prosthetic heme group, which is crucial for maintaining its enzymatic activity. In general, standard laboratory strains have limited capacity to uptake heme from the extracellular environment. Different approaches have been designed to overcome this issue and increase heme content in cells (Varnado et al., 2004). We assumed that permeabilization of bacterial membranes or extracellular production could have a positive impact on homogeneous incorporation of heme into the active site of protein and therefore ensure high activity. For instance, *E. coli* strain RP523 with introduced uncharacterized permeability mutations was heme-permeable when grown on heme supplemented medium (Li et al. 1988). However, no significant differences were observed in the catalase or peroxidase activity of AfKatG and the peroxidase activity of MDBP in permeabilized cells or samples isolated directly from growth medium (Tables 3 and 4). Thus, this approach may provide an alternative for production of other heme proteins with homogeneous heme content and activity.

We also evaluated the capability of our system by expressing other model proteins such as DNA polymerases. Native thermostable DNA polymerases are usually expressed at low levels by thermophilic bacteria. *E. coli* or baculovirus production systems were used as hosts for production of active forms of several DNA polymerases. *Pfu*, *Pwo* and *Taq* were produced in *E. coli* at expression levels of approximately 24 mg/L, 26.6 mg/L and 95 mg/L, respectively (Dabrowski et al., 1998; Engelke et al. 1990). *Pfu* polymerase was also produced into growth medium using *Spodoptera frugiperda* cells (sf-9) and *Trichoplusia ni* cells (hi-5) with final yields of 100 mg/L and 134 mg/L, respectively (Mroczkowski et al. 1994). However, despite their ability to achieve higher production quantities, baculovirus systems are more expensive and more challenging to manipulate compared to bacterial systems. We have used *V. natriegens* Vmax for the production of wild type *Taq* polymerase into the growth medium, achieving final yields of 26.5 ± 6.0 mg/L (Table 5). The abundance of the soluble Mut*Taq* polymerase in intracellular fraction was significantly lower compared to the wild-type enzyme when expressed in both *V. natriegens* strains. However, Mut*Taq* polymerase was an interesting model protein for the application of the PBP5/6 co-expression system due to its high insolubility when produced intracellularly in *V. natriegens* Vmax (Obr. 7) or in *E. coli* (data not shown). The intracellular fraction of Mut*Taq* polymerase without D,D-carboxypeptidase PBP5/6 remained insoluble, probably due to high protein concentration. However, we have purified approximately 10.4 ± 5.6 mg/L of Mut*Taq* polymerase directly from medium using the PBP5/6 co-expression strategy. However, both *Taq* and Mut*Taq* polymerase lacked enzyme activity when purified from the intracellular fraction or directly from the growth medium (See supplementary material). The loss of the polymerisation activity could be the result of chosen method for protein precipitation, protein degradation, or longer timeframe of cultivation (24 h), which is however necessary to reach sufficient yield of model proteins in the growth medium. This limitation could indicate a drawback of our approach.

Targeting recombinant proteins into the growth medium offers several important advantages over intracellular strategies. When a protein is transported directly into growth medium, it remains in its soluble form and reduces negative effects on the bacterial culture and overall production. This approach simplifies the purification process since no cell disruption is required and overall number of purification steps can be reduced while maintaining the same level of product purity (Quax 1997). The downstream process often includes many purification steps to reach the highest purity, resulting in significant loss of valuable material. Production of recombinant proteins directly into growth medium is one of the strategies to reduce the number of required purification steps and overall process costs (Yoon et al. 2010).

In this study, we demonstrated the effect of D,D-carboxypeptidase PBP5/6 overexpression on extracellular leak of model proteins, such as GFP, AfKatG, MDBP and *Taq*/Mut*Taq* polymerase into the growth medium. As we assumed, co-expression of D,D-carboxypeptidase had a positive impact not just on the abundance of model protein in growth medium but also on purity of these model proteins after chromatography when purified directly from medium. This procedure can be highly beneficial for downstream process optimization, both at laboratory scale and in high-level protein production.

## Conclusions

The approach of D,D-carboxypeptidase overexpression could provide the access for intracellular proteins into growth medium without the use of specific secretion signals, fusion tags, additional supplements, or overexpression of secretory machinery and without extensive loss of production capacity of the cell. Nonspecific leakage of intracellular proteins enhanced by overexpression of D,D-carboxypeptidases PBP4 and especially PBP5/6, without significant culture deprivation, could provide a useful way to transport recombinant proteins into media in *V. natriegens*. Overexpression of D,D-carboxypeptidase PBP5/6 had a positive impact on the transport of intracellular proteins such as GFP, AfKatG, MDBP or *Taq polymerase* (Mut*Taq*) into growth medium. This simple production strategy could also provide acceleration of downstream processes, reduction of overall production costs, and a promising alternative for production of recombinant proteins with high production rate as well as low solubility in *V. natriegens* strains.

### Electronic supplementary material

Below is the link to the electronic supplementary material.

Supplementary Material 1

## Data Availability

All data generated or analysed during this study are included in this published article (and its supplementary information files).
