# Supplementary material for: Novel expression system based on enhanced permeability of Vibrio natriegens cells induced by D,D- carboxypeptidase overexpression
Source: World J Microbiol Biotechnol. 2023 Aug 12;39(10):277. doi: 10.1007/s11274-023-03723-z (PMC10421817; doi:10.1007/s11274-023-03723-z)
Supplement: Supplementary file 1 — Supplementary Material 1 [file 11274_2023_3723_MOESM1_ESM.docx]

**Novel efficient recombinant expression system based on enhanced permeability of *Vibrio natriegens* cells induced by D,D-carboxypeptidase overexpression**

**Ľubica Kormanová^1^, Zdenko Levarski^1,2^, Andrej Minich^1^, Viktor Varga^1^ Lenka Levarská^2^, Eva Struhárňaská^1^, Ján Turňa^1^, Stanislav Stuchlík^1^**

^1^ Faculty of Natural Sciences, Comenius University in Bratislava, Ilkovičova 6, Bratislava 811 04, Slovak Republic; [kormanova10@uniba.sk](mailto:kormanova10@uniba.sk) (L.K); [zdenko.levarski@uniba.sk](mailto:zdenko.levarski@uniba.sk) (Z.L); [minich5@uniba.sk](mailto:minich5@uniba.sk) (A.M); [varga121@uniba.sk](mailto:varga121@uniba.sk) (V.V); [struharnans1@uniba.sk](mailto:struharnans1@uniba.sk) (E.S); [jan.turna@uvp.uniba.sk](mailto:jan.turna@uvp.uniba.sk) (J.T); [stanislav.stuchlik@uniba.sk](mailto:stanislav.stuchlik@uniba.sk) (S.S)

^2^ Science Park, Comenius University in Bratislava, Ilkovičova 8, Bratislava 811 04, Slovak Republic. [lenka.levarska@uvp.uniba.sk](mailto:lenka.levarska@uvp.uniba.sk) (L.L); [zdenko.levarski@uniba.sk](mailto:zdenko.levarski@uniba.sk) (Z.L)

* Correspondence: [kormanova10@uniba.sk](mailto:kormanova10@uniba.sk)

**Supplementary material**

1. *Construction of recombinant plasmids*

The genes *dacA* and *dacB* representing D,D-carboxypeptidases PBP4 and PBP5/6 respectively were amplified from *V. natriegens* ATCC 14048 genome by PCR with specific primers (Tab 1.). Primers were designed with restriction sites for enzymes *BglII* and *PvuI* (*dacA*) and *BamHI* and *NotI* (*dacB*) for subsequent DNA cloning. Q5 high fidelity DNA polymerase (NEB) was used for amplification with annellation temperatures of 61 °C (*dacA*) and 63 °C (*dacB*) and appropriated thermocycling conditions. Plasmid pRSFDuet-ADH was digested with restriction enzymes *BglII*/*PvuI* (NEB) for restriction cloning of *dacA* (PBP5/6) for creation of pRSFDuet-T7-PBP4. For construction of pRSFDuet-T7-PBP5/6, we have used plasmid pRSFDuet-ADH digested with restriction enzymes *BamHI*/*NotI*-HF (NEB). PCR fragments for *dacA* (PBP4) and *dacB* (PBP5/6) were also digested with restriction enzymes *BglII*/*Pvu*I and *BamHI*/*NotI*-HF, respectively. Prepared fragments for each recombinant construct were ligated using T4 DNA ligase (NEB) and transformed into *E. coli* DH5α.

The T7 promoter in previously prepared recombinant constructs pRSFDuet-T7-PBP4 and pRSFDuet-T7-PBP5/6 was replaced with hybrid T5-*lac* promoter which was amplified by PCR using specific primers (Supplementary material) from pJexpress404-AfkatG as template. Amplified T5-*lac* fragment and pRSFDuet-T7-PBP4 were digested with restriction enzymes *NotI*-HF/*BglII* (NEB) and ligated using T4 DNA ligase (NEB), resulting in recombinant construct pRSFDuet-T5-PBP4. Afterwards, the *dacB* (PBP5/6) gene was amplified using specific set of primers with restriction sites for *BglII* /*XhoI* (NEB). *DacB* PCR product and pRSFDuet-T5-PBP4 construct were digested with *BglII*/*XhoI* (NEB) restriction enzymes. Prepared fragments were ligated using T4 DNA ligase (NEB) and transformed into *E. coli* DH5α.

We have also prepared a recombinant construct with GFP gene under T5-*lac* promoter. Vector fragment was amplified by specific primers with added sites for restriction enzymes *EcoRI*/*XbaI* (NEB) from template (pJexpress404-AfkatG). Plasmid pJK100-T7-GFP was digested with *EcoRI*/*XbaI* enzymes (NEB) and appropriate fragment of interest was isolated from agarose gel after separation of digested products. DNA fragments were ligated using T4 DNA ligase (NEB) and *E. coli* DH5α stain was transformed with these ligation products. All the prepared recombinant constructs were subsequently analysed and confirmed by restriction digestion, PCR, or sequencing.

**Table 1** Bacterial strains and vectors used in this work.

| **Plasmids** | **Basic characteristic** | **Source** |
| --- | --- | --- |
| pRSFDuet-ADH | T7-promoter, RSF origin, Amp^R^ | (Utekal et al. 2014) |
| pRSFDuet-T7-PBP4 | T7-promoter, RSF origin, Cm^R^ | This work |
| pRSFDuet-T7-PBP5/6 | T7-promoter, RSF origin, Cm^R^ | This work |
| pJK100-T7-GFP | T7-promoter, ColE1 origin, Amp^R^ | (Krahulec el al. 2010) |
| pJexpress404-T5-GFP | T5-promoter, ColE1 origin, Amp^R^ | This work |
| pRSFDuet-T5-PBP4 | T5-lac promoter, RSF origin, Cm^R^ | This work |
| pRSFDuet-T5-PBP5/6 | T5-lac promoter, RSF origin, Cm^R^ | This work |
| pJexpress404-AfkatG | T5-lac promoter, ColE1 origin, Amp^R^ | (Struharnanska et al. manuscript in preparation) |
| pET28a-T7-Taq | T7-promoter, pBR322 Kn^R^ | ongoing research |
| pET28a-T7-MutTaq | T7-promoter, pBR322, Kn^R^ | ongoing research |
| pJexpress404-T5-MDPB | T5-promoter, ColE1 origin, Amp^R^ | ongoing research |
| **Strains** | **Genotype** | **Source** |
| *E. coli* DH5α | supE44 ∆(lacZYA-argF) U196 (Φ80∆lacZM15) hsdR17 recA1. endA1 gyrA96 thi-1 relA1. | Invitrogen |
| *V. natriegens* Vmax™ | Major extracellular  nuclease knockout, insertion of an IPTG-inducible T7 RNA polymerase cassette | Synthetic Genomics® |
| *V. natriegens* PF | Δvpn1 Δvnp2 | (Pfeifer et al. 2017) |
| *V. natriegens* 14048™ | *V. natriegens* wild type | ATCC 14048™ |

**Table 2** Sequence of primers used for construction of recombinant plasmids.

| **Used primers** | **Sequence** |
| --- | --- |
| PBP4 ch1-F (*BglII*) | *GGCCAGATCTatgcgtttacgttggtcc* |
| PBP4 ch1-R (*PvuI*) | *GGCCGATCGctacttcttaggcatcgcctg* |
| PBP 5/6 ch1-F (*BamHI*) | *GGCCGGATCCatgaataaaaataagtttgtgaaatctattctc* |
| PBP 5/6 ch1-R (*NotI*) | *GGCCGCGGCCGCttagaagaagctcttaactaacaatac* |
| T5-F (*NotI*) | GGCCGCGGCCGCAGCTTCATGCACAGTGAAATCATG |
| T5-R (*BglII*) | GGCCAGATCTTTTTACCTCCTAAAAGTTAAACAAAATTATTTC |
| T5-PBP 5/6 (*BglII*) | GGCCAGATCTatgaataaaaataagtttgtgaaatctattctc |
| T5-PBP5/6 (*XhoI*) | *GGCCCTCGAGttagaagaagctcttaactaacaatac* |
| AfkatG-F | ATGATGCGTCAGGGTGG |
| AfkatG-R | CTCGAGGCGCAGACCTGC |
| T5-GFP-F (*EcoRI*) | GGCCGAATTCGACCCCAAGGGCGACACC |
| T5-GFP-R | ATGTTTTACCTCCTAAAAGTTAAACAAAATTATTTCTAG |
| GFP-F | GATGAGTTGTTTAAATGCTAGCAAAGGAGAAGAAGAACTTTTCAC |
| GFP-R | GGGGGAATTCTTATTTGTAGAGCTCATCCATGCATGTG |

1. *Recombinant plasmids used in this work.*


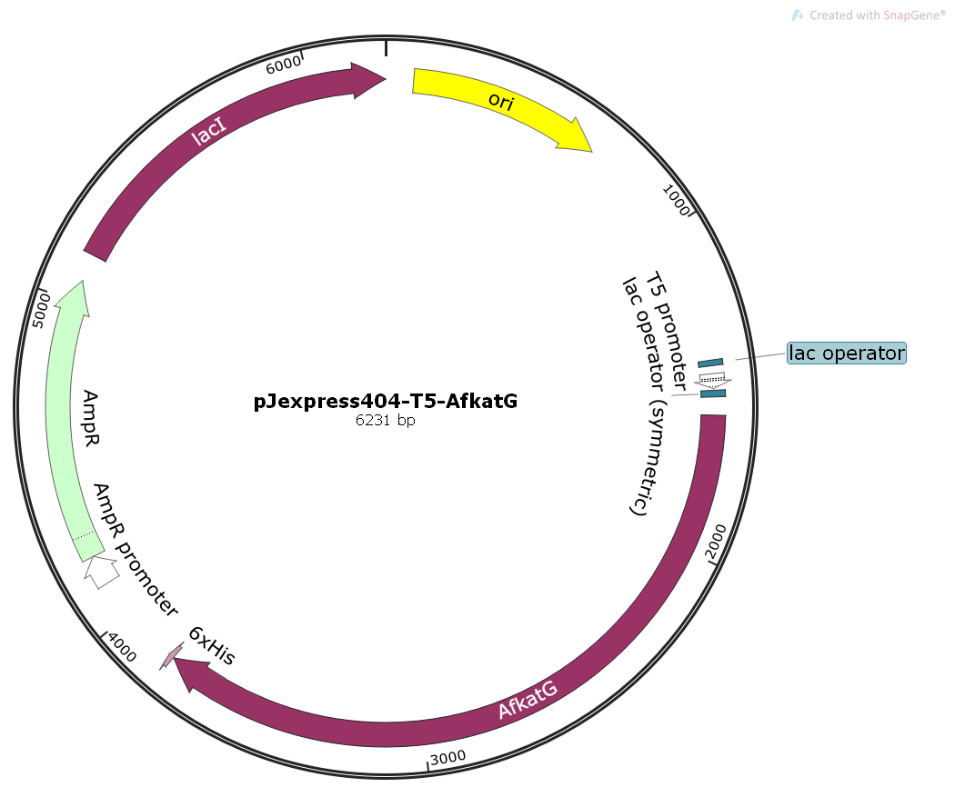


**Fig. 1** Recombinant plasmid for AfkatG production under T5-*lac* promoter and its basic characteristics (Struharnanska et al. manuscript in preparation).


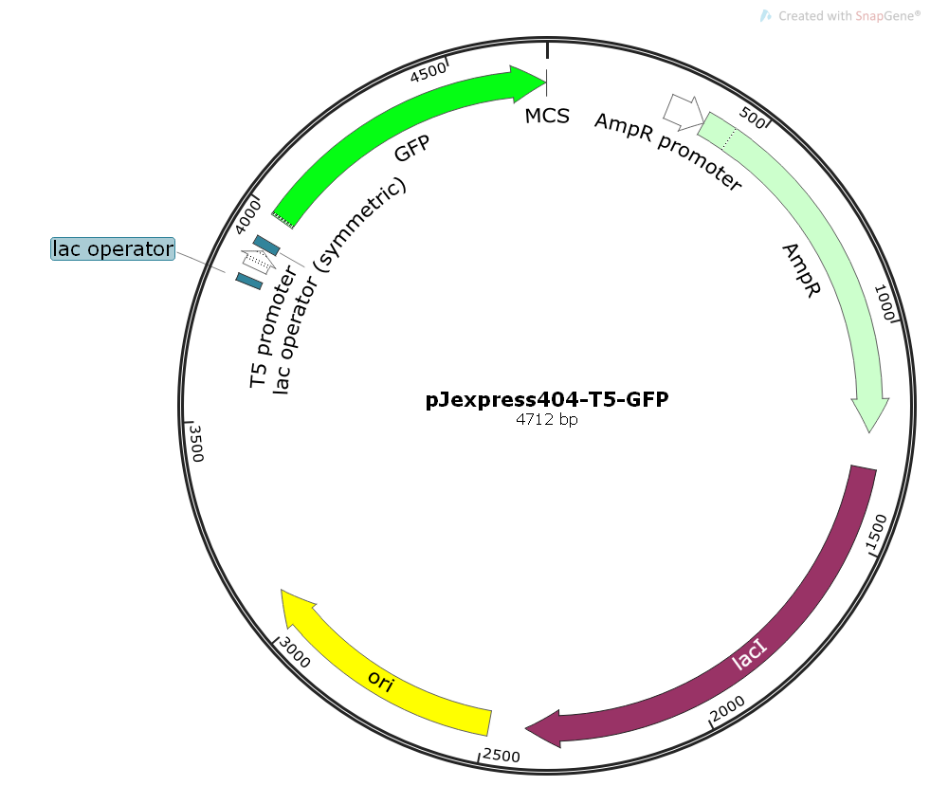


**Fig. 2** Recombinant plasmid for GFP expression under T5-*lac* promoter and its basic characteristics.


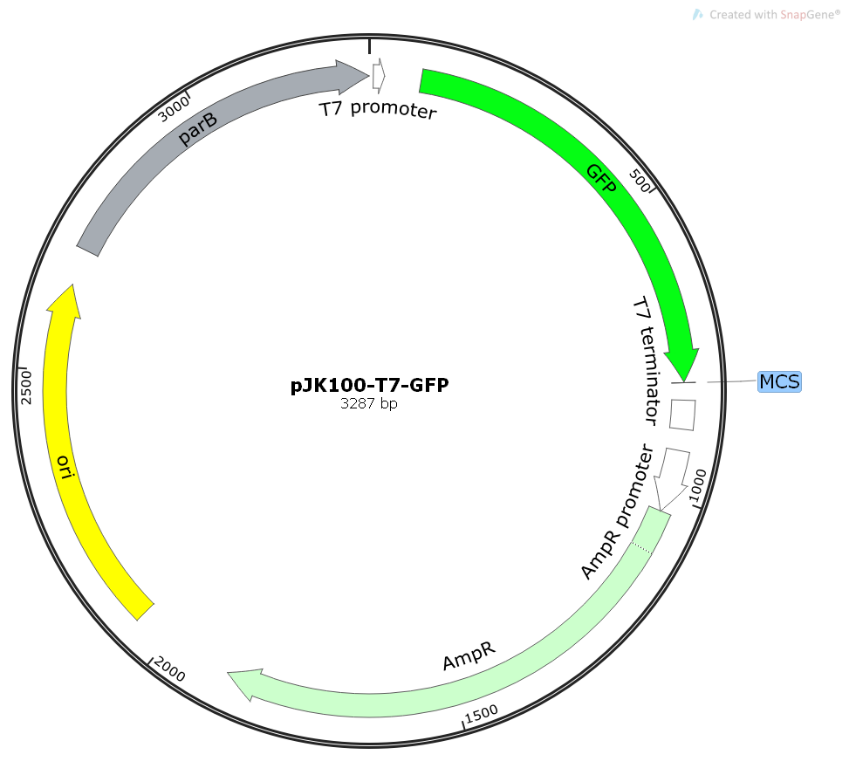


**Fig. 3** Recombinant plasmid for GFP expression under T7 promoter and its basic characteristics (Krahulec el al. 2010).


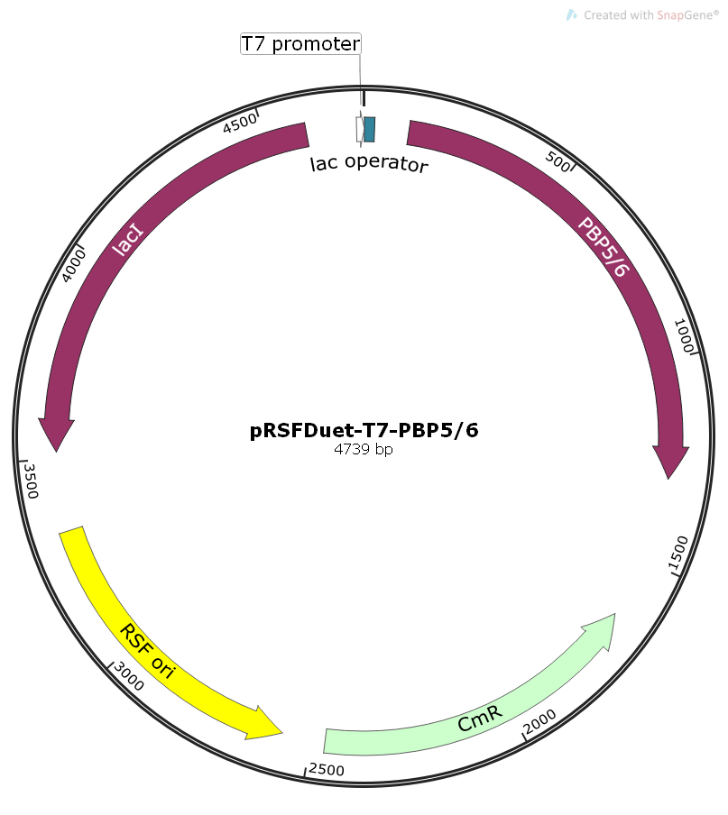


**Fig. 4** Recombinant plasmid for PBP5/6 carboxypeptidase overexpression under T7 promoter and its basic characteristics.


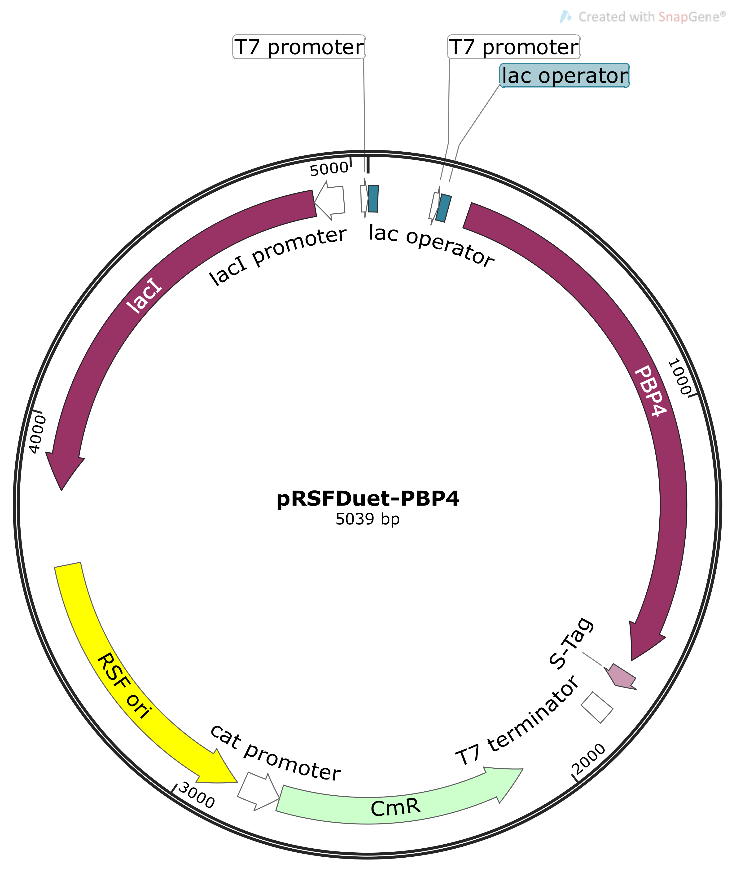


**Fig. 5** Recombinant plasmid for PBP4 carboxypeptidase overexpression under T7 promoter and its basic characteristics.


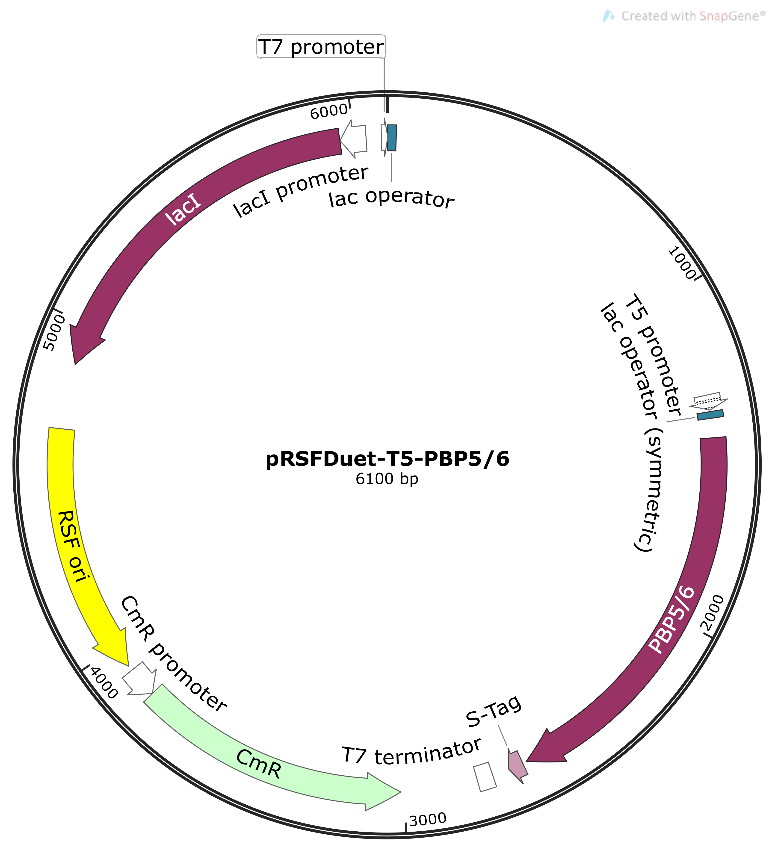


**Fig. 6** Recombinant plasmid for PBP5/6 carboxypeptidase overexpression under T5-*lac* promoter and its basic characteristics.


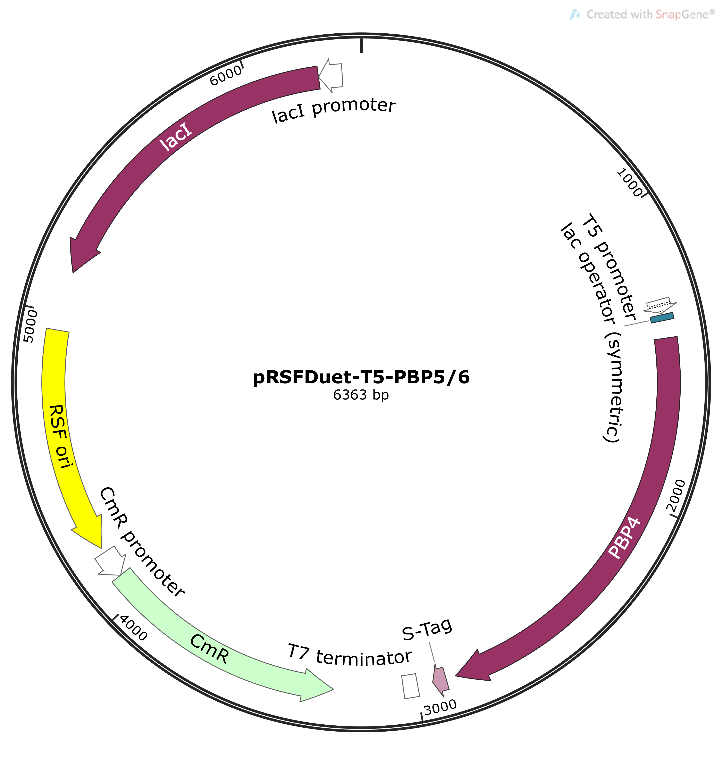


**Fig. 7** Recombinant plasmid for PBP4 carboxypeptidase overexpression under T5-*lac* promoter and its basic characteristics.


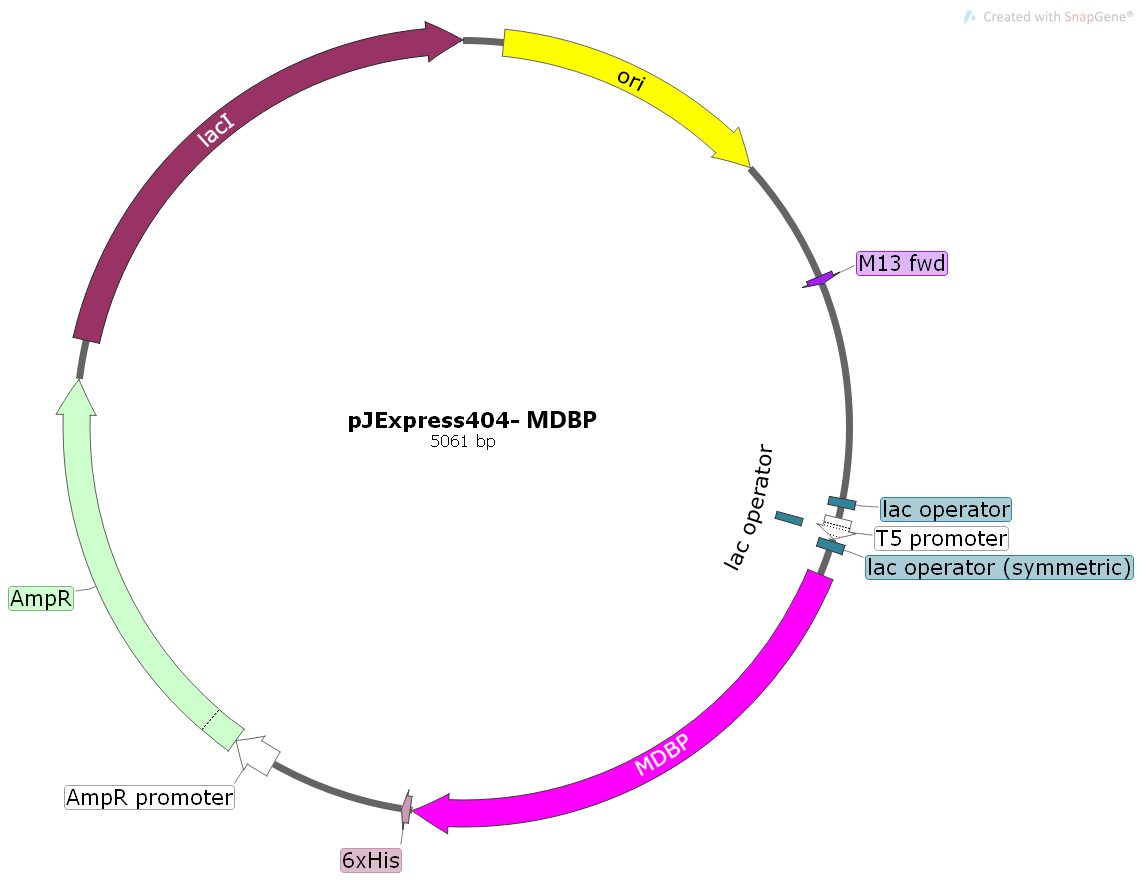


**Fig. 8** Recombinant plasmid for MDBP peroxidase expression under T5-*lac* promoter and its basic characteristics.


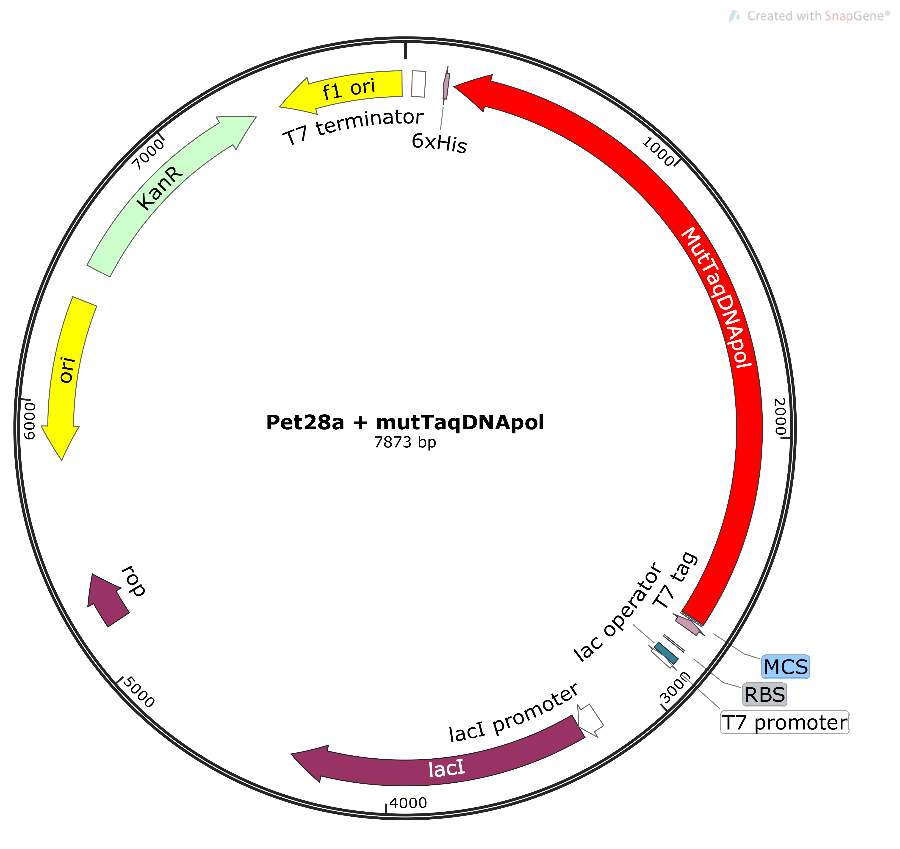


**Fig. 9** Recombinant plasmid for Mut*Taq* polymerase overexpression under T7 promoter and its basic characteristics.


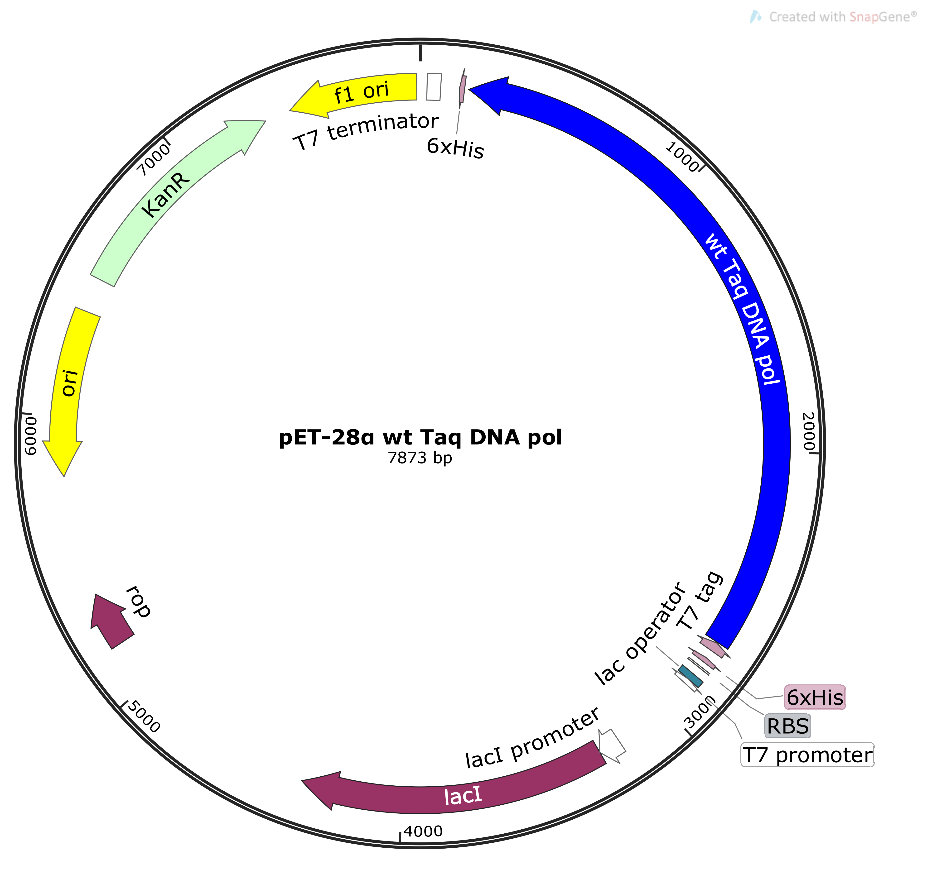


**Fig. 10** Recombinant plasmid for wild type of *Taq* polymerase overexpression under T7 promoter and its basic characteristics.

1. *Enzyme activity of model proteins*

After IMAC chromatography of *Taq* and Mut*Taq* polymerases, no enzyme activity was observed when purified from medium or cells (Fig. 11).


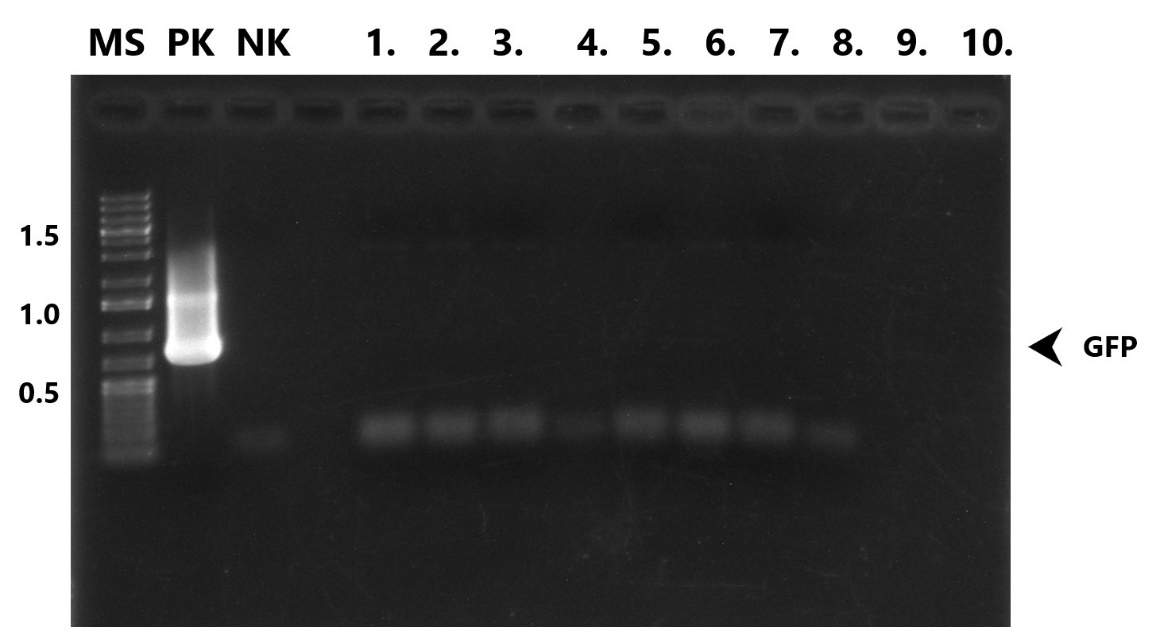


**Fig. 11** Agarose electrophoresis of PCR products amplified using commercial *Taq* polymerase (Thermo Scientific ™), produced wild type Taq polymerase and Mut*Taq* polymerase in *V. natriegens*. Wild type *Taq* polymerase – intracellular production (1.-3.); Wild type *Taq* polymerase – extracellular production (4.-6.), Mut*Taq* polymerase – intracellular production (7.); Mut*Taq* polymerase – extracellular production (8.-9.); PK – positive control (commercial *Taq* polymerase); NK- negative control.





**Fig. 13** Determination of specific catalase and peroxidase activity of AfkatG produced intracellularly or extracellularly using approach with co-expression of PBP5/6 carboxypeptidase. Enzyme activity was determined from elution samples after purification using IMAC; A – Specific peroxidase activity; B – Specific catalase activity; ns – non-significant; A *p*-value less than 0.05 was considered statistically significant. IP – intracellular protein; EP – extracellular protein.


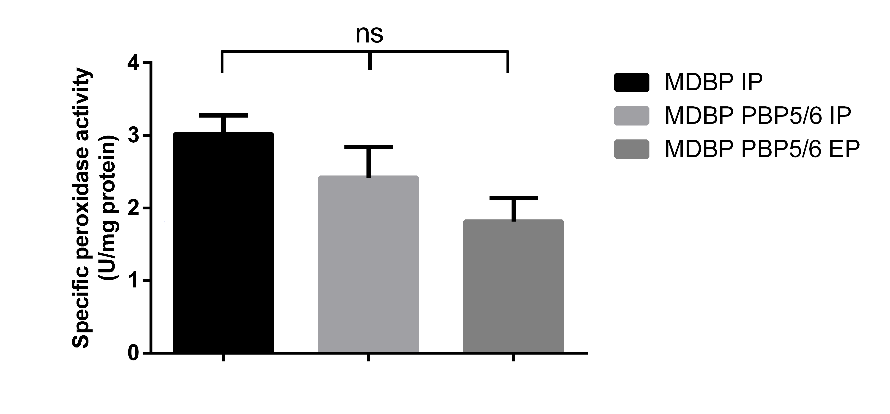


**Fig. 14** Determination of specific peroxidase activity of MDBP produced intracellularly or extracellularly using approach with co-expression of PBP5/6 carboxypeptidase. Enzyme activity was determined from elution samples after purification using IMAC; A *p*-value less than 0.05 was considered statistically significant. ns – non-significant; IP – intracellular protein; EP – extracellular protein.

1. *Determination of total protein abundance in growth medium*

**
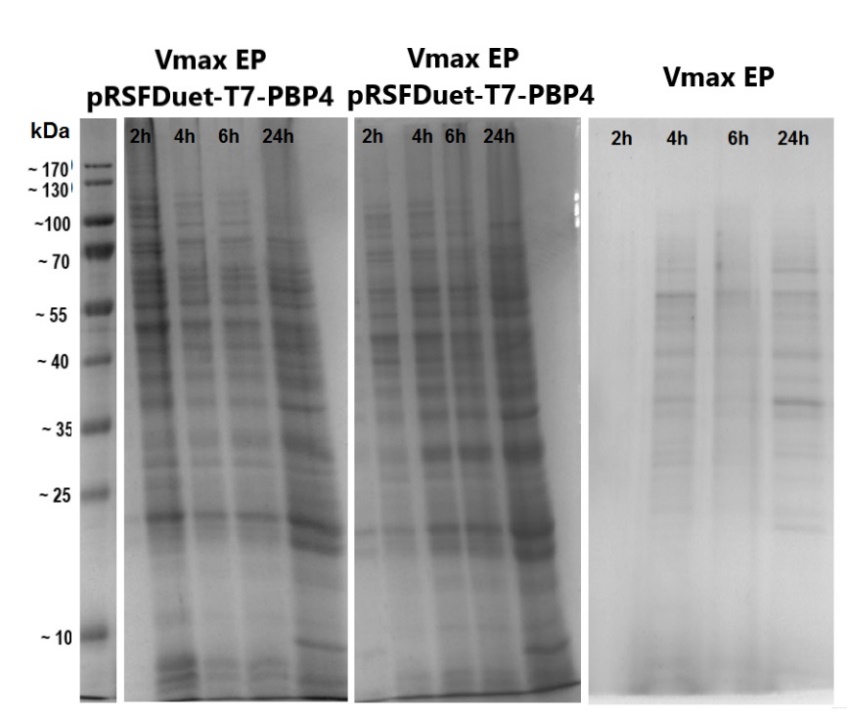
**

**Fig. 15** **A** - SDS-PAGE analysis of total protein in growth medium after D,D-carboxypeptidase PBP4 or PBP5/6 overexpression under T7-inducibile promoter in *V. natriegens* Vmax. Control cultivation is without overexpression any of the enzymes. IP – intracellular protein; EP – extracellular protein.


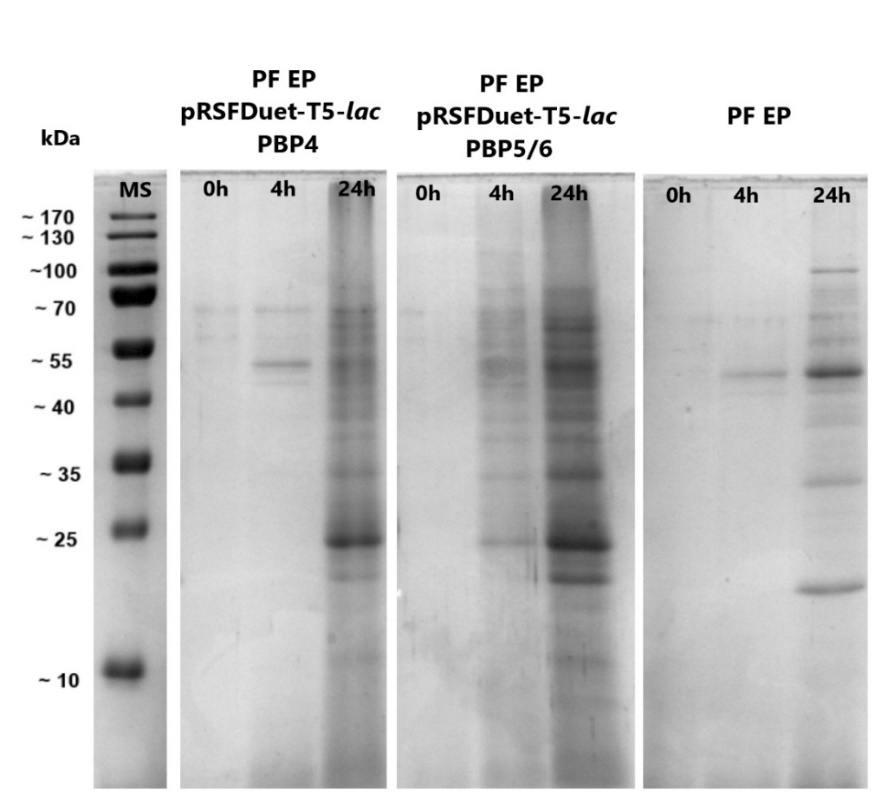


**Fig. 16** – SDS-PAGE analysis of total protein in growth medium after D,D-carboxypeptidase PBP4 or PBP5/6 overexpression under T5- lac promoter in V. natriegens PF. Control cultivation is without overexpression any of the enzymes. IP – intracellular protein; EP – extracellular protein. Control cultivation is without overexpression any of the enzymes. IP – intracellular protein; EP – extracellular protein. PF – *V. natriegens* Prophage-free

**

**

**Fig. 17** Analysis of the total protein abundance in growth medium (after 4h and 24 h) D,D-carboxypeptidase overexpression using Bradford method. **A**- Overexpression of D,D-carboxypeptidase PBP4 or PBP5/6 under T7 promoter in *V. natriegens* Vmax. **B** – Overexpression of D,D-carboxypeptidase PBP4 or PBP5/6 under T5-*lac* promoter in *V. natriegens* PF. Control cultivation is without overexpression any of the enzymes. PF – *V. natriegens* Prophage-free

1. *Determination of specific growth rate in V. natriegens*





**Fig. 18** Determination of specific growth rate (in exponential growth phase) in *V. natriegens* cultivations harbouring overexpressed D,D-carboxypeptidase PBP4 or PBP5/6. In control cultivation was present no D,D-carboxypeptidase overexpression.

**References**

Krahulec J, Hyrsova M, Pepeliaev S, Jilkova J, Cerny Z, Machalkova. J (2010) High level expression and purification of antimicrobial human cathelicidin LL-37 in *Escherichia coli*. Appl Microbiol Biotechnol 88(1):167–175

Pfeifer E, Michniewski S, Gätgens C, Münch E, Müller F, Polen T, Millard A, Blombach B, Frunzke J, Stabb EV (2019) Generation of a Prophage-Free Variant of the Fast-Growing Bacterium *Vibrio natriegens*. Applied and Environmental Microbiology 85(17):00853-19

Utekal P, Tóth C, Illésová A, Koiš P, Bocánová L, Turňa J, Drahovská H, Stuchlík S (2014) Expression of soluble *Saccharomyces cerevisiae* alcohol dehydrogenase in Escherichia coli applicable to oxido-reduction bioconversions. Biologia 69(6):722–726. <https://doi.org/10.2478/s11756-014-0376-6>
